# Development of Conductivity Sensors for Multi-Phase Flow Local Measurements at the Polytechnic University of Valencia (UPV) and University Jaume I of Castellon (UJI)

**DOI:** 10.3390/s17051077

**Published:** 2017-05-10

**Authors:** José Luis Muñoz-Cobo, Sergio Chiva, Santos Méndez, Guillem Monrós, Alberto Escrivá, José Luis Cuadros

**Affiliations:** 1 Instituto de Ingeniería Energética, Universitat Politècnica de València, 46022 Valencia, Spain; aescriva@iqn.upv.es (A.E.); jocuaor@upv.es (J.L.C.); 2 Unidad de Mecánica de Fluidos, Universitat Jaume I, 12071 Castellón, Spain; schiva@emc.uji.es (S.C.); gmonros@emc.uji.es (G.M.); 3 Faculty of Electric and Mechanical Engineering, Universidad Autónoma de Nueva León, Monterrey 66451, Mexico; santos.mendezdz@uanl.edu.mx

**Keywords:** multi-sensor conductivity probe, conductance sensors, sensors for two-phase flow

## Abstract

This paper describes all the procedures and methods currently used at UPV (Universitat Politécnica de Valencia) and UJI (University Jaume I) for the development and use of sensors for multi-phase flow analysis in vertical pipes. This paper also describes the methods that we use to obtain the values of the two-phase flow magnitudes from the sensor signals and the validation and cross-verification methods developed to check the consistency of the results obtained for these magnitudes with the sensors. First, we provide information about the procedures used to build the multi-sensor conductivity probes and some of the tests performed with different materials to avoid sensor degradation issues. In addition, we provide information about the characteristics of the electric circuits that feed the sensors. Then the data acquisition of the conductivity probe, the signal conditioning and the data processing including the device that have been designed to automatize all the measurement process of moving the sensors inside the channels by means of stepper electric motors controlled by computer are shown in operation. Then, we explain the methods used for bubble identification and categorization. Finally, we describe the methodology used to obtain the two-phase flow information from the sensor signals. This includes the following items: void fraction, gas velocity, Sauter mean diameter and interfacial area concentration. The last part of this paper is devoted to the conductance probes developed for the annular flow analysis, which includes the analysis of the interfacial waves produced in annular flow and that requires a different type of sensor.

## 1. Introduction

This paper describes the main activities and results at the Institute of Energy Engineering of the Polytechnic University of Valencia (UPV), in collaboration with the Fluid Dynamic Unit of the University Jaume I of Castellon (UJI), related to the design, use, manufacture and performance obtained using different types of conductivity sensors for two-phase flow, and conductance sensors for film flows.

Three fundamental parameters—void fraction, Sauter mean diameter, and interfacial area concentration (IAC)—characterize the basic structure of a two-phase flow [1]. The void fraction expresses the phase distribution and we need it for the hydrodynamic and thermal design in various industrial processes. On the other hand, the interfacial area describes the available area for the interfacial transfer of mass, momentum and energy, and it is a fundamental parameter for any two-fluid model formulation, and the key for most of the industrial applications of two-phase flows. Various transfer mechanisms between the phases depend on the two-phase interfacial structures. Therefore, an accurate knowledge of these parameters is necessary for any two-phase flow analyses.

The non-intrusive methods available to measure those parameters have limited use. Optical methods, like PIV, LDA, or CCD cameras are useful when the void fraction is low and the parameters to be measured do not change too fast with time [2,3]. Other methods like electrical tomography are quite complex to process when one needs good spatial resolution [4]. Ultrasound, gamma ray or X-ray attenuation techniques are quite expensive and nowadays may not have good spatial resolution, although they are much more accurate in determining mean values such as fractional flow velocity (up to 3%) or void fraction (up to 10%) [5,6]. An interesting and accurate new method is imaging by nuclear magnetic resonance, the main inconvenience being that it is expensive. A well stablished intrusive method to measure multi-phase flow parameters is the use of needle probes. Different measuring techniques are available for these needle probes. The techniques based on conductivity, capacitance or optical measurements, are the most popular ones. Based on the phase identification, these probes can provide information about void fraction, IAC, bubble size, frequency, and velocity using simple physical principles. The most extensively used probes for intrusive local measurements are optical or conductivity ones. Both probe typologies are very similar and they share the same signal processing methodology with minor changes. The number of the sensors employed in each probe has increased during recent years, and probes with two, four (Kim [7]), five (Euh [8]), or six (Shen [9]), sensors have been proposed with good results, although in general, increasing the number of probe tips beyond four does not improve the results very much.

The main goal and motivation of this research during the last few years was to develop corrosion resistant multi-sensor conductivity probes, and to establish a clear methodology that provides good values for the parameters that characterize the two-phase flow in vertical pipes starting from the sensor raw signals. To confirm that both sensors and methodology give good experimental results for the two phase-flow parameters, we performed a set of cross comparisons with experimental data obtained by other authors using the same boundary conditions as explained later in this paper. In addition, we measured the two-phase flow parameters by different methods to confirm the results obtained with the conductivity probes. A secondary goal was to obtain a database for two-phase flow, in vertical pipes, covering different two-phase flow regimes and conditions and the transitions between them [10,11,12,13]. To achieve these objectives it was necessary to develop a set of multi-sensor conductivity probes, to select the best ones, and from the signals of these sensors (tips) to obtain the information on the local characteristics of the two-phase flow parameters, void fraction, bubble velocity, Sauter mean diameter, interfacial area concentration and so on. The starting point were the previous works performed by Kataoka, Ishii and Serizawa [14], Kim, Fu, Wang and Ishii [15], Revankar and Ishii [16], Wu and Ishii [17], Shen et al. [18], and many others.

The first step in this work was to build the sensor, but the first sensors assembled in our laboratories degraded very fast with time and after only a few measurements it was necessary to replace them, so it was necessary to manufacture these sensors in such a way as to make these sensors resistant to corrosion. We explain with some detail in this paper in Section 2.1 the way to build these sensors. We tested many different materials and coatings and after many trials, we found the optimal ones for the intended sensor design. In addition, it was necessary to design an electric circuit for the conductivity probe. To achieve this goal, we tried two types of circuits, in the first one the signal acquisition from the different tips was in parallel, but in this case, there was an electric coupling between the signals provided by the different sensors of the probe. Therefore, we decided that each sensor should have an independent circuit, as explained in Section 2.2 of this paper.

In addition, the operation and data acquisition with these sensors it is very important, as explained in Section 2.3. The sensors are introduced into the experimental channel by means of a device called a port. At first, we tried to perform the measurements manually by inserting the sensor with a device moved with the help of a micrometric screw, but this method was too lengthy. Therefore, we designed a system that manipulated the insertion of the sensor conductivity probe automatically by means of a stepper electric motor controlled by a computer.

Another important issue is the signal conditioning and processing, i.e., how the signals are manipulated in order that the useful information can be extracted from them. This is explained in Section 2.4. Section 2.6 discusses the degradation of the sensors caused by corrosion and by deposits, showing how the sensor signal behaves depending on the degradation type, and displaying several examples of signals obtained with degraded sensors.

Section 2.7 and Section 2.8 analyze the problem of bubble identification and categorization. Bubbles are classified in different types or groups depending on their characteristics. The method used is based on the measured chord length that it is obtained from the sensor signals as explained in Section 2.7. Then, taking into account previous works of Ishii [19], Kim et al. [15] and Mendez [20], the bubbles are classified according to the chord lengths.

One important question when using multi-sensor conductivity probes in two-phase flow measurements is the methodology employed to obtain the values of the physical magnitudes of the flow from the sensor signals. Section 3 shows first how to obtain the local void fraction from the sensor signals. Then in Section 3.2 and Section 3.3 how to obtain the bubble velocity from the signals of the multi-sensor conductivity probe, and how to correct the measured velocity using calibration factors are explained. Finally, Section 3.4 and Section 3.5 show the methods used to obtain the interfacial area concentration (IAC) from the sensor signals. In Section 3.6, we give the flow conditions for some experiments and the results obtained using the previous methodology, then we discuss the characteristics of these results and if they are consistent from a physical point of view. Then, in Section 3.7, we show some experimental results obtained with two kinds of conductivity probes, and in Section 3.8, we provide the results obtained to verify that the experimental methods designed with the multi-sensor conductivity probe give results that are similar to the ones obtained using different experimental methods. In addition, a cross comparison has been performed using the same boundary conditions as in another experiments performed by other authors to see if the results were the same.

Another area in which we are working, and that we explain briefly in Section 4, is the development of sensors for annular flow. The types of sensors that have been built are conductance probes for the measurement of the film thickness in annular flow. The reason is that annular flow is one of the most important regimes in the steam generation systems currently used to produce electricity (coal, nuclear and biomass power plants) and it could be present in about 70% of the length of the channels where two-phase flow is produced. The heat transfer coefficient of this type of flow is very high, and it depends on the thickness of the liquid film, so it is very important to know the characteristics of the interfacial waves that the steam flow produces on the liquid film, so the sensors must be designed with this goal in mind. These waves are related to the droplet entrainment and to the dry-out of the pipe wall, and to analyze this phenomenology, non-intrusive technical measurements are recommended because they do not affect the liquid film. In the liquid film, there are two types of waves: the disturbance waves, which have big amplitude, and the ripple waves, which have less amplitude than the disturbance ones. The main focus of our study are the disturbance waves, because in the crest of these waves droplets are produced due to the dragging effect of the gas flow, being then entrained by the steam flow. The thickness of the waves affects the heat transfer coefficient. In the literature, we can find two non-intrusive methods or techniques to measure the liquid film thickness: optic methods and electric methods. The electric technique was selected to measure the time evolution of the liquid film in the annular flow produced in our laboratory [21,22]. With both methods, the two types of waves in annular flow can be studied, and the main parameters, such as wave amplitude, form, frequency and length, can be measured. Nevertheless, the electric methods have the advantage that the measurements can be performed in case of bad visibility. In our experimental conditions, we have a surface with curvature, cylindrical geometry, that distorts the images and, in addition, we have droplets so we cannot put borescopes inside the experimentation channel to observe and video-record these waves because the drops and the mist make very difficult to take good pictures.

## 2. Measurement of the Void Fraction, the Interfacial Area Concentration and the Velocity Using Two and Four Tip Conductivity Probes

### 2.1. Building of the Conductivity Probes

**Figure 1:**
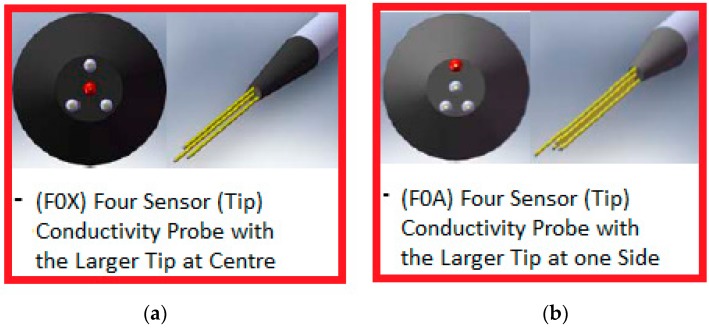
Types of four sensor conductivity probes used to measure the interfacial area concentration, the void fraction distribution and the bubble velocity. (**a**) Sensor with the larger tip at the center; (**b**) Sensor with the larger tip at one side

**Figure 2:**
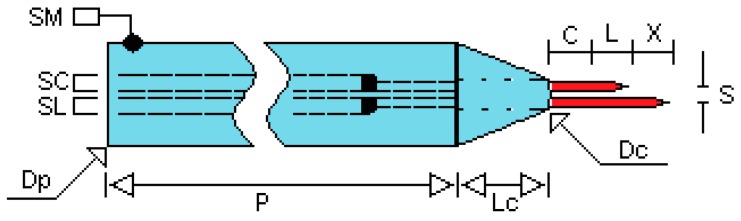
Two tips conductivity probe built in at UPV and UJI.

A conductivity probe is an intrusive instrument designed for two-phase flows measurements that can have normally from two up to five tips, or conductivity sensors arranged in a given geometric distribution and that allow to detect the change in conductivity that takes place at each tip location, when the two-phase fluid moves through the tips. Figure 1 displays some examples of the four tips conductivity probes manufactured in our laboratories, denoted as F0X and F0A, the first with the larger tip in the center and the second with the larger tip at a side. In addition, several two sensor (tips) conductivity probes were built that are shown at Figure 2. Each conductivity sensor or tip is located at the extreme of a needle coated with an isolation material, and the sensing part is at the extreme and it is coated with a conducting material, which should be resistant to corrosion.

Table 1 lists the geometric characteristic of these conductivity probes. We denote by SM, SC and SL the electric terminals that allow the connection of the conductivity probe to the electric circuit. The distance between the two tips, denoted as (X) in Figure 2, was fixed to 1.5 mm ± 0.2 mm in some prototypes and to 2.2 mm ± 0.2 mm in others and must be performed with great precision. The separation between both needles, denoted by (S), was maintained constant by means of a ceramic tube of the type of the ones used in chromatography and that have very small internal channels inside.

The main problem found during the manufacture of the conductivity probes was the fast degradation and corrosion of the conductivity probe tips and the isolation material that covers the needles. Because the most sensible part is the sensor/tip, the first design was oriented to choose the isolation material of the needle and to select the tip material coating. The selected needles were made of stainless steel in all cases. The diameter and length of these needles without coating was [∅, l] = [0.35 mm, 150 mm]. We also tested stainless steel needles coated with silver, with [∅, l] = [0.32 mm, 150 mm], and stainless steel needless coated with gold [∅, l] = [0.26 mm, 40 mm]. In the three cases, the response to corrosion was good.

Another important fact in the design of the conductivity probes was the isolation material that cover the needles and that must have a good dielectric behavior. An important property in the evaluation of this isolation material is the difficulty in the application of this material to the needle surface and its layer by layer deposition. The selected material should be easy to apply and form layers, which should be resistant to degradation by corrosion and hard enough to withstand the probe assembly process. Urethane provided the best results; it showed a good resistance to corrosion and good dielectric material properties. The application method to form the layers consisted in submerging the stainless steel needle in urethane and then slowly withdrawing the needle covered with a layer of coating and wait for some time until the deposited layer had dried. Then the needle was submerged again in the urethane to deposit a new layer. We repeated this process until we had added three dielectric layers. After that, the coated needle was kept for four hours inside an oven at 60 °C.

**Figure 3:**
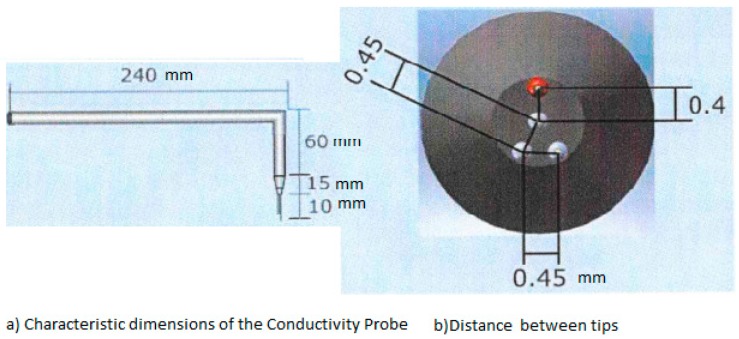
Characteristic dimensions of the four tips conductivity probes F0A used by UPV and UJI to measure the interfacial area concentration and the bubble velocity in bubbly flow regime, and the transition from bubbly to cap/slug regime.

The distance between the large tip and the short tips was chosen between 1.5 and 2 times the expected diameter of the bubbles, then for bubbly flow and the observed diameter of the bubbles, we used a distance of 2.2 mm. As displayed in Figure 1a,b, the shorter tips form an equilateral triangle of 0.45 mm each side, while the larger tip (red) is located at 0.4 mm from the nearest tip in the radial direction, see Figure 3 for more details.

Figure 3a displays the main geometric dimensions of the four tips conductivity probe used in the experiments performed at UPV to measure the radial distribution of the interfacial area concentration and void fraction. The horizontal length was set to 240 mm and the automatic positioning system was assembled on this horizontal part of the conductivity probe, while the vertical part with a length of 60 mm (see Figure 3) was used to avoid disturbing the two-phase flow with the positioning system. This vertical part was oriented in the direction of the flow pointing downward in the vertical channel. Thus, the bubbles touched first the large or reference tip of the probe.

Finally we must say that, as shown in Figure 2, the needles in the two sensors conductivity probe merge from a cylinder that terminates in a cone made of an epoxy polymer material that prevented the inflow of fluid to the stainless steel cylinder where the lower part of the needles were located. This material is made of two components that when mixed become harder and harder during the curing process. Finally, the conical shape was shaped by hand at the beginning of the drying process when the epoxy mixture was still soft enough.

### 2.2. Electric Circuit of the Conductivity Probe

**Figure 4:**
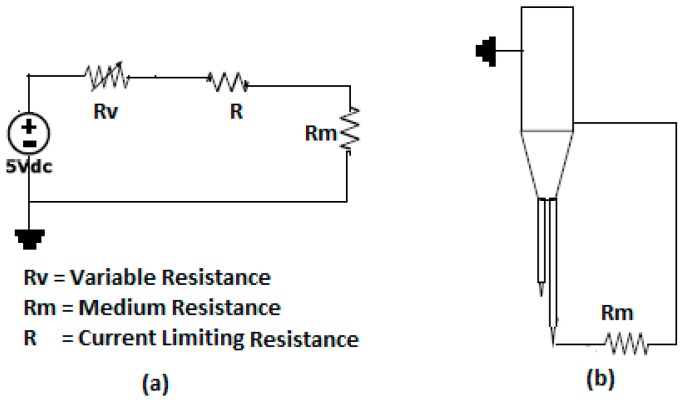
Prototype of the electric circuit used in the tips of the conductivity sensors. (**a**) Electric circuit for the tips, (**b**) Electric resistance of the fluid medium for the larger tip.

The goal of the electric circuit is to provide the current intensity and voltage needed by the conductivity probe and, at the same time, to condition the signal in such a way that the data acquisition system receives a good quality signal. In order to have a good performance of the electric circuit it is necessary to use a highly stable and precise laboratory power-supply source. In addition, it was necessary to have an electric circuit for each one of the conductivity probe tips. Figure 4 displays the prototype of the electric circuit used in the measurements.

The electric resistance *R* displayed at Figure 4 has a value of *R* = 100 kΩ and has been designed to limit the current in the circuit. The variable resistance $R_{V}$ = 0–100 kΩ was designed to adjust the output signal to a given predetermined value, and the resistance $R_{m}$ was the resistance produced by the fluid medium (gas or liquid). The previous circuit was mounted inside a polypropylene box with selected electric connectors to avoid any potential damage during its connection to the acquisition data cards. Table 2 displays the results of the electric measurements when changing the fluid medium from water to gas, the goal was to quantify the value of the electric resistance of the fluid water or gas. In ideal operating conditions, we must have:

(1) $$V=R_{T}I=\left(R+R_{V}+R_{m}\right)I$$

(2) $$R_{m}=\frac{V}{I}-\left(R+R_{V}\right)$$

The results of the experimental measurements showed that the air electric resistance was approximately 500 times bigger than the electric resistance obtained when the fluid was water.

Another important issue was the amount of noise in the signal provided by the conductivity probe. Most of the signal noise was due to the supply source noise; to reduce the amount of noise we used a laboratory stabilized power supply source with reduced noise. This source was adjusted to provide 5 Vdc with a maximum intensity of 0.02 mA, and 2% output tolerance, with maximum voltage ripple of 7 mV. All the signal voltages were referred to the reference of the data acquisition system. The voltage and resistance measurements were performed with a multi-meter Yokogawa TY530 series with accuracy of ±(0.09% reading+2 digit) for the voltage measurements and ±(1% reading+1 digit) for the resistance measurements below 0.01 MΩ and ±(1% reading+2 digit) for the resistance measurements above 0.01 MΩ, and the intensity was calculated not measured.

### 2.3. Operation and Data Acquisition of the Conductivity Probe

**Figure 5:**
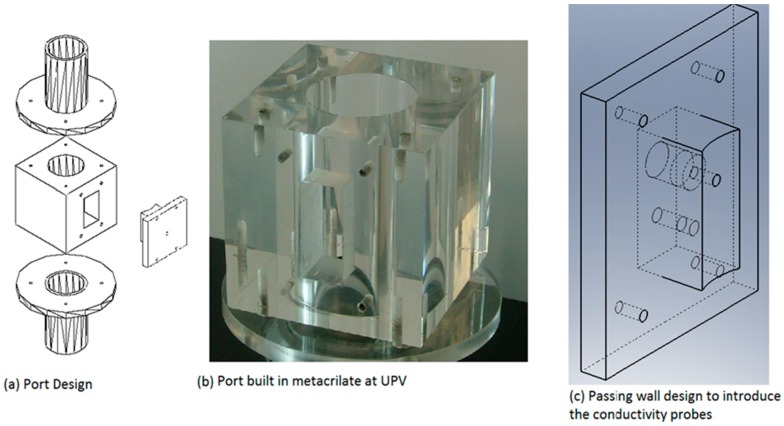
Design of the “port” used to place the conductivity probe in the test section and used in the experiments performed at the PUMA facility at UPV.

The conductivity probe was mounted inside the test channel with the needles parallel to the flow direction pointing downward, after that the three sensor terminals, for the two-sensor conductivity probe, were connected and the electric circuit was feed by the power supply with 5 Vdc, and a data acquisition frequency of 50 kHz is recommended. In order to correctly position the conductivity probe in the test section of the channel, it was necessary to design a device, described as a “port”, to bring the sensor into the test section avoiding fluid leakages and perturbing the two-phase flow as less as possible. Figure 5 displays this device.

Once we inserted the conductivity probe into the measured test section by means of the port, we needed to displace it radially to perform the measurements at different distances from the pipe center. At first, we performed the measurements positioning the conductivity probe manually. However, we discarded this procedure because each measurement for one flow condition was performed at 15 radial positions and three axial ones and the amount of time needed to position the conductivity probes correctly was too large. Therefore, we decided to build an automatic positioning system for the conductivity sensors by means of a device that contained a stepper electric motor controlled by a computer. The automatic measurements were performed simultaneously at three different axial locations, using three computed controlled devices that radially moved three conductivity probes. Each positioning device contained one stepper electric motor controlled by computer, the user provided a length to the computer and the computer sent a pulse train to a control card that feed the stepper motor coil until the desired position was attained. The precision of this positioning device was 0.025 mm.

The analog signals obtained from the conductivity probes were between 2 V and 5 V, and one channel of the data acquisition card was set up for each sensor tip of the conductivity probe. A NI-Max card (National Instruments, Austin, TX, USA) with a resolution of 16 bits and with a total maximum sampling rate of 200,000 samples/s was used. A LABVIEW (National Instruments) program controlled the data acquisition and signal processing to be explained in the next subsection. The LABVIEW program contained a subroutine that stored the conductivity probe radial positions, where the measurements were to be performed. These positions were transmitted to the automatic positioning devices, and at each radial position, the signal coming from the conductivity probe was captured and, then, the results were stored in a text file. This text file contained the following information: the electric signal from each tip, the sampling rate, and the number of samples acquired by each sensor. When the data coming from the last position were acquired, then the program ordered to the positioning device of the conductivity probe to return to the initial position and to wait for a new set of flow parameters conditions.

### 2.4. Filtering, Conditioning and Binarization of the Signals

**Figure 6:**
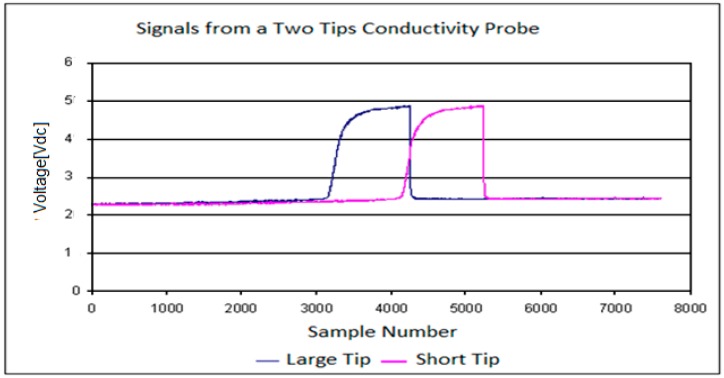
Signals produced at the larger and short tips of a two-sensor conductivity probe installed in PUMA facility at UPV.

The four and the two tip conductivity probes provided information about the residence time of each phase in the sensors and the time interval that needed each bubble to travel between the point where it touched the large tip and the point where it touched one of the shorter tips. To obtain this useful information it was necessary to perform the signal processing that consisted in the following four steps: (i) signal conditioning, (ii) classification of the bubbles in groups, (iii) evaluation of the flow parameters, (iv) averaging and correction. Figure 6 displays the signals obtained from the two-tip conductivity probe before the signal conditioning; we observe a time delay between the front (large) and rear (short) sensor signals, due to the time needed by the bubble to travel from one sensor to the other one. Also for each signal the time of residence of each phase in each sensor is clearly observed in Figure 6.

**Figure 7:**
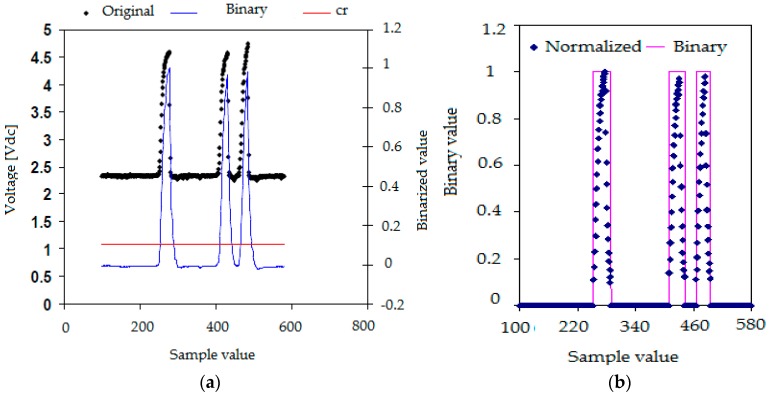
(**a**) Signal trains previous and after the normalization process and threshold value (red), (**b**) Binary (red) and normalized (blue points) signal trains.

The extraction of the information contained in each one of the registered signals began by performing a moving average filtering to eliminate the high frequency noise produced by the electronic acquisition system, in this way we obtained smoothed signals. Once this filtering was finished, we performed a normalization of the signal values so the signal values after normalization (blue lines) belong to the interval [0,1] as displayed in Figure 7a (right scale). This normalization was achieved as follows, first, for each conductivity probe tip k, we obtained the maximum $V_{\max,k}$ and minimum $V_{\min,k}$ values of the signal voltages (*k* = 0, 1, 2, 3). Then, we calculated the normalized signal values for each tip k of the conductivity probe by means of the following expression:

(3) $$V_{i,k,N}^{\prime }=\frac{V_{i,k}-V_{\min,k}}{V_{\max,k}-V_{\min,k}}$$

where $V_{i,k,N}^{\prime }$ denoted the *i*-th normalized signal value belonging to the *k*-th tip signal, $V_{i,k}$ denoted the i-th signal voltage value, finally $V_{\min,k}$ and $V_{\max,k}$ denoted the minimum and maximum values respectively of the electric potential measured at the *k*-th tip, with *k* = 0, 1, 2, 3. Then for each tip *k*, we set a threshold value denoted by $cr_{k}$ and that is the red line in Figure 7a This last figure displays the original signal measured in one tip after the median filtering step (black points), the normalized one (blue) and the threshold value (red line). The mission of the threshold was to remove small variations of the voltage when the tips detected only liquid, therefore all the points at tip *k* with electric voltage smaller than the threshold for the *k*-th tip were removed. Then several measurements were performed in order to determine the most convenient value for the threshold voltage $V_{threshold}$ = $cr_{k}$, we arrived to the conclusion that a 10% of the signal amplitude, 0.1 in the normalized scale of the right hand side of Figure 7a, was enough to remove these small fluctuations of the signals. This threshold value was then added to the normalized signal bottom and all the signal fluctuation smaller than the resulting value were removed.

The criterion used to detect the beginning of a bubble interface was to count the number of signal data points with voltage above the threshold value. Then, if we had less than five points, over the threshold value, with increasing values (positive increments) of the voltage that followed a point *i* with voltage equal or below the threshold value, then we decided that the tip was detecting the beginning of a bubble and we set the binarized value of the signal to 1. If it happened that we did not have five points with increasing values of the electric potential then we considered that we were detecting electric noise and the value was set to zero. Figure 7b displays the binarization process for a signal. Proceeding in the same way, we defined the back interface of the bubble when we had five consecutive points following a given point with voltages different from zero and with decreasing values of the electric voltage, as displayed in Figure 7b.

### 2.5. Experimental Facility

**Figure 8:**
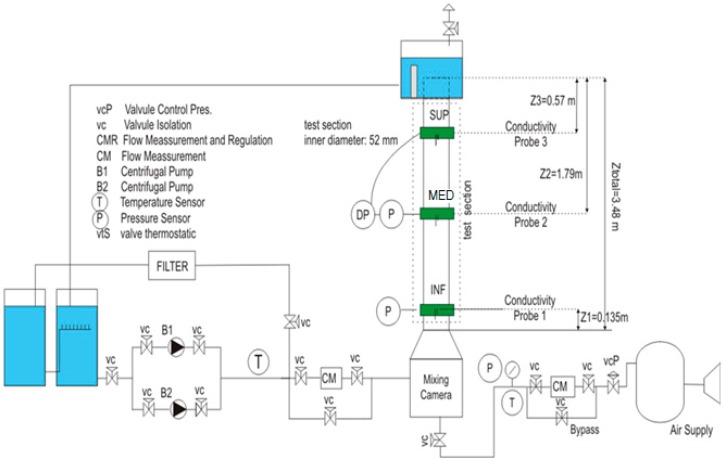
Layout of the facility used to perform the two-phase flow experiments.

Figure 8 displays the outline of the experimental facility used to perform the experimental work. It is a thermo-hydraulic loop, with a test section, a lower plenum where the air and the water are mixed, and an upper plenum where it takes place the separation of the gas and liquid phases. The test section is a round transparent tube made of Plexiglas^®^ with constant section, inner diameter of 52 mm, and length of 3340 mm. We use purified water as working fluid that circulates moved by two centrifugal pumps controlled by a frequency controller. A compressor supplies air to the test section. The air pass to the test section through a porous sinter element with an average pore size of 10 mm installed below the mixing chamber at the lower plenum. The air and the water temperatures were kept constants during the test assays. The air mass flow rate was measured by two thermal mass flow meters and controllers (EL-FLOW model, range 5–200 L/min, range 50–1400 L/min, Bronkhorst^®^, Ruurlo, Netherland), and the liquid flow rate by a electromagnetic flow meter (Badger Meter^®^, Milwaukee, WI, USA, range 0–70 m^3^/h). The probe measurements were performed at three axial locations, Z/D = 2, 36 and 56, named port INF, port MED and port SUP respectively. At each port we measured the pressure by a PTX 600 series type precision pressure transmitter (GE DRUCK^®^, Boston, MA, USA) at port INF with range [0, 1] bar, and at port MED and SUP with range [0, 250] mbar and with a precision of 0.5%.

In order to measure the liquid velocity we have used Laser Doppler Anemometry (LDA). The LDA equipment consists of a 0.5 W Ar+ laser (Omnichrome-Dantec, Skovlinde, Denmark) a Fiberflow beam separator (Dantec, Skovlunde, Denmark) Dantec FVA 58N40 processor and one PC using the Floware software for data acquisition. We used a lens of 0.125 m focal length, operating the system in the backscattering mode. The vertical component was determined with green (λ = 514.5 nm) beams and the horizontal component with blue (λ = 488 nm) beams. A pre-shift frequency of 500 kHz was used. The flow was seeded with hollow particles, which were neutrally buoyant with a 10 μm mean size (Dantec HGS-10); therefore, only the liquid velocity was measured.

### 2.6. Corrosion and Degradation of the Conductivity Probes

**Figure 9:**
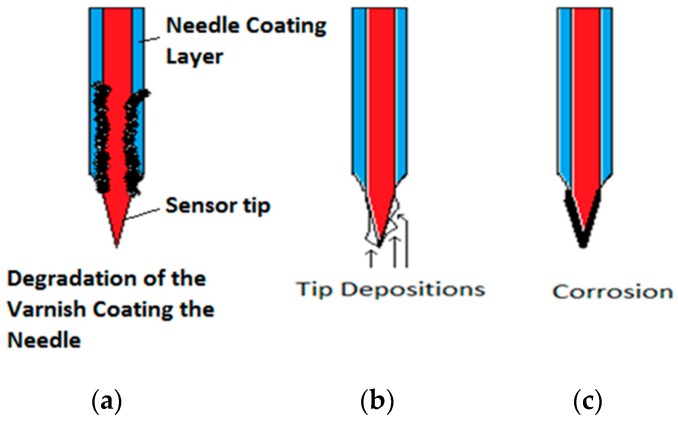
Different degradation mechanisms of the sensors of the conductivity probe. (**a**) Example of degradation of the varnish coating, (**b**) Example of tip depositions, (**c**) Example of tip corrosion.

A very important issue from a practical point of view is to manufacture conductivity probes that degrade as slowly as possible. In this subsection, we study the degradation of the sensors of the conductivity probe and the signals obtained when the tips or the needle isolation degraded. The capability to manufacture conductivity probes that were resistant to corrosion was a critical issue because the isolating coating of the needle, as well as the metal of the tips, degraded with time due to the contact with the water. The sensor (tip) also suffered electrochemistry degradation because the sensors were fed with an electric current. Figure 9 displays the different degradation mechanisms of the conductivity probe that we observed during its use. The degradation starts with a progressive separation of the deposed layer that covers the needle near the tips. Another observed degradation mechanism was the appearance of deposits on the tip, these deposits changed the electric resistance, increasing its value, and therefore modified the sensor response. In addition, these deposits retained some water and the signal value diminished when the sensor was in contact with the gas phase.

**Figure 10:**
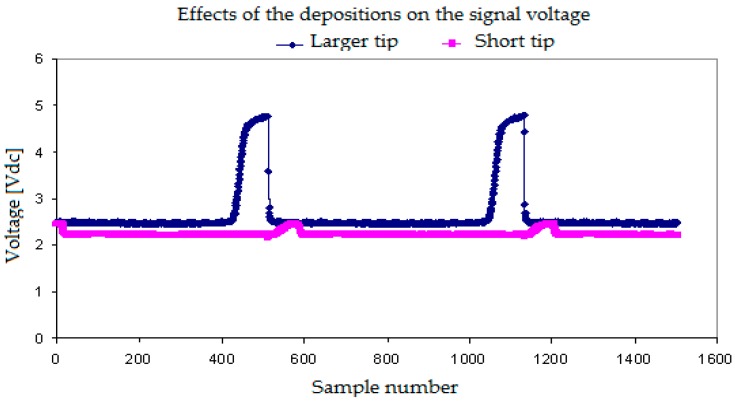
Signal degradation by depositions on the short sensor of the two tips conductivity probe.

Figure 10 displays the effect of the deposits on the short tip signals when we used the two tip conductivity probe. It is observed that, in this case, the increase of the electric resistance in addition to the effect that this deposit had retaining some water and diminishing the electric potential signal value when the gas phase touched the tip. It is observed in Figure 10 that the signal coming from the short tip is smaller than the signal coming from the front tip due to the short sensor degradation.

**Figure 11:**
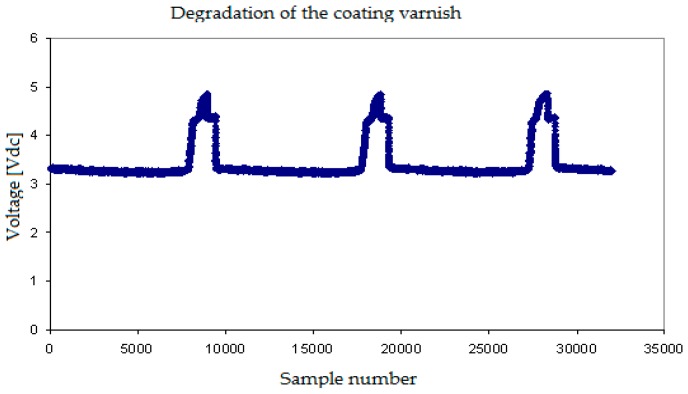
Effect on the sensor signal produced by the degradation of the varnish layer deposed on the needles.

Finally, we display some results obtained when the tip of the conductivity sensor was affected by degradation of the isolating layer covering the needle, that was partially lost in some points and also by the simultaneous corrosion of the tip. In this case, we observed (Figure 11) that the maximum and minimum voltages provided by the sensor when touching the gas and water phases respectively were different from normal sensors. The minimum signal value was higher, and the difference between the maximum and the minimum values of the signal become smaller. In addition, the signals displayed smaller secondary peaks.

### 2.7. Bubble’s Identification with the Four and Two Sensors (Tips) Conductivity Probe

**Figure 12:**
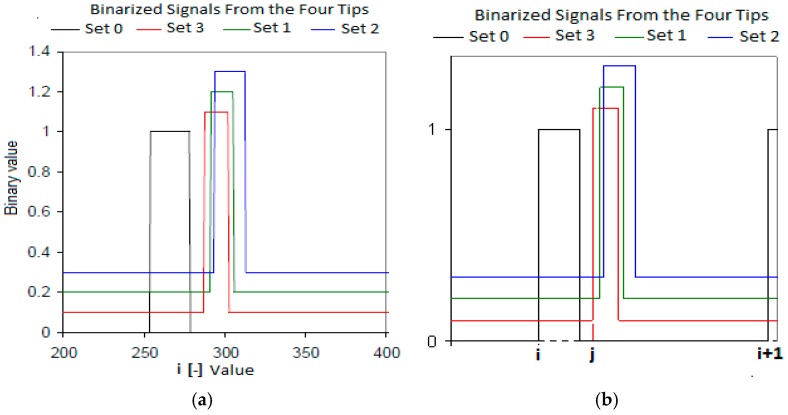
Binarized signals from the four tips conductivity probe.

The first step was to detect the initial and end times for each bubble in the four tips conductivity probe, a similar process holds for the two tips conductivity probe. Figure 12 displays an example of the binary signals provided by the four-sensor conductivity probe assuming that during its motion the bubble touched the four tips with its front and back interfaces. Then we proceeded to the bubble identification by searching in the binary signals of the four tips. During the identification process, the larger tip was considered as the main reference, in such a way that we identified in this signal the beginning of the *i*-th bubble, denoted by $B_{i}$, and the beginning of the *i*+1 bubble, denoted by $B_{i+1}$, as it is displayed in Figure 12b. This process of identification of the initial time for each bubble in the front sensor (larger one) is known as the interface-pairing signal process [11,12,15,18]. After setting the initial time in the larger tip, for instance for bubble *i*, we looked in the shorter tips, which were located downstream, for the signals produced by this bubble at those tips. If we denote by $B_{j}$ the initial time of the *j*-th bubble squared signal detected by one of the shorter sensors located downstream. Then if this signal *j* is generated by the same bubble that produced the *i*-th squared signal in the front sensor (larger tips), then to belong to the same bubble the following condition should be met:

(4) $$B_{i}<B_{j}<B_{i+1}$$

Then we obtained the time difference $\Delta B_{i-j}=B_{j}-B_{i}$ between the signals provided by both tips. However, to be completely sure that the bubble that generated the *i*-th signal in the reference sensor was the same one that produced the *j*-th signal in the shorter tip, we estimated first the interfacial velocity using a very simple drift flux model [11,12]. Then, on account that the distance among the sensors was known, we obtained an estimation of the time of flight $\Delta t_{est-fr}=\Delta S_{k}/v_{drif-flux}$, from the front to the rear sensors. Then, to consider that the same bubble produced the signal *j*, it should be in the interval $\left[0.8\Delta t_{est-fr},\;1.2\Delta t_{est-fr}\right]$, also we looked for one signal in the rest of the rear sensors. Finally, if we found that all the rear sensors had signals that belonged to this range, then we considered that the bubble signal was “paired”, and we considered that we have an effective bubble.

Therefore, one signal was considered as effective when the bubble touched the four sensors in the logic order, i.e., first the reference sensor located upstream, and then the three rear sensors located downstream of the reference sensor. However, due to the turbulence and to the radial motion some bubbles did not touch some of the rear sensors, in this case when some of the rear signals was missed we said that the bubble was “missing”.

The next step was to look for at each rear sensor signal, not paired with a signal in the front sensor, its corresponding pairs in the other rear sensors, these signals were denoted as non-paired rear sensor signals. This operation was performed estimating the time interval needed by the bubble that touched one of the rear sensors to travel to the rear sensor that it was farther away, and considering a 20% margin. During this process, both directions were considered, i.e., that the bubble was coming to this rear sensor or that the bubble was moving away to the other sensors. The signals paired in the rear sensor but not in the front sensor were assigned to the same missing bubble. At the end we got all the bubble effective signals and all the missing bubble signals. The determination of a factor that takes into account all the missing bubbles is crucial for the determination of a reliable value of the interfacial area concentration and other local two-phase flow parameters.

### 2.8. Bubble Categorization

**Figure 13:**
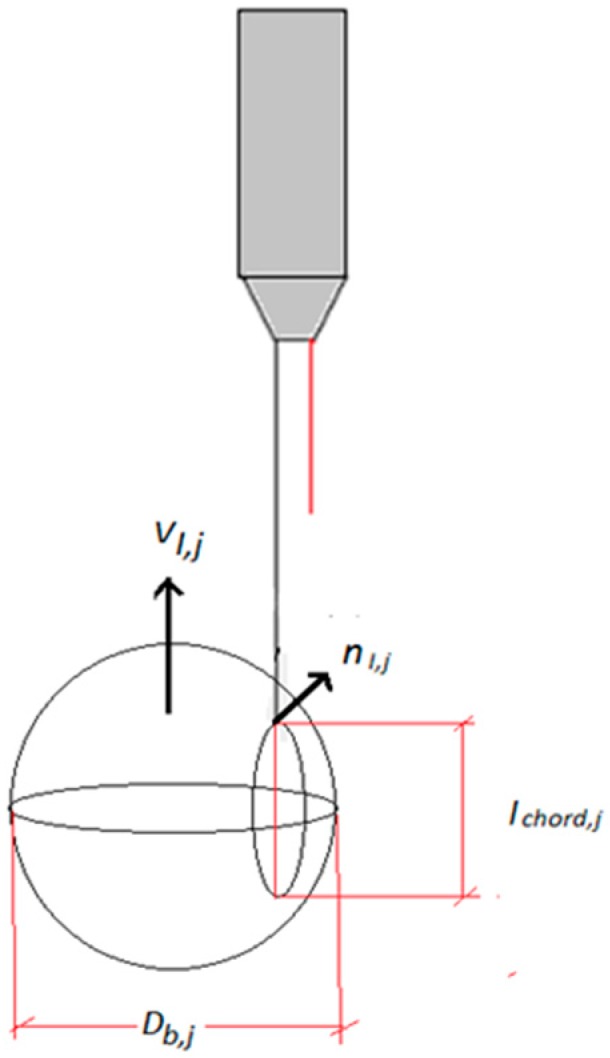
Chord length $l_{chord,j}$ for a bubble passing with velocity $V_{I,j}$ through the reference tip of a two sensors conductivity probe.

When we consider not only bubbly flow formed by small spherical bubbles but also cap/slug flow and the transition regime from bubbly to cap/slug flow, the problem of the bubble categorization or classification of the bubble signals according to the type of bubble (spherical, distorted, cap, slug) which have produced each recorded signal becomes very important. To solve this problem we need a criterion that can classify the bubble signals according to bubble type. This problem was discussed and studied by Ishii et al. [19] and Kim et al. [15] using a criterion based on the chord length obtained from the reference sensor signal for each bubble. Because, according to the previous section, we measured the initial and final residence time for each bubble in the reference sensor. In addition, it is easy to measure the velocity of each bubble interface, as will be explained in the forthcoming sections, dividing the distance between the large sensor and one of the short sensors by the time of flight needed by the bubble to travel between them. Therefore, one can estimate the so-called chord length of each bubble displayed in Figure 13, and denoted by $l_{chord,j}$, as the length obtained when we multiply the time that the gas-phase for a given bubble remains in the reference sensor by the velocity measured for this bubble.

Therefore, based on the bubbles chord-length, it is possible to classify the bubble signals and whether a given bubble belongs to a certain type of bubble or to another one. This process, called categorization, has been performed following the categorization criteria of Ishii [19], Kim et al. [15], and Mendez [20]. The bubbles are classified according to their chord lengths into four groups: spherical, distorted, cap, and slug. The limits for these groups are:

1. Spherical bubbles if the chord length $l_{chord,j}$ belongs to [0, $D_{spherical,\;\max}$
2. Distorted spherical bubble if $l_{chord,j}$ belongs to [$D_{spherical,\;\max}$, $D_{distorted,\;\max}$]
3. Caps bubbles if $l_{chord,j}$ belongs to [$D_{distorted,\;\max}$, $D_{caps,\max}$]
4. Slugs if $l_{chord,j}>D_{\sec tion}$

where the group limits are defined by the following expressions:

(5) $$D_{spherical,\;\max}=5.65\;D_{ref}N_{\mu_{f}};\;D_{distorted,\max}=4\;D_{ref};\;D_{caps,\max}=40\;D_{ref}$$

where the reference bubble diameter $D_{ref}$ is taken equal to the Laplace length, and $N_{\mu }$ is the viscosity number. Both parameters are given by the following expressions, respectively:

(6) $$D_{ref}=\sqrt{\frac{\sigma }{g\Delta \rho }}\;and\;N_{\mu_{f}}=\frac{\mu_{f}}{{\left(\rho_{f}\sigma D_{ref}\right)}^{1/2}}$$

where σ is the surface tension, $\mu_{f}$ is the dynamic viscosity, and $\Delta \rho =\rho_{f}-\rho_{g}$ the density difference. To have an idea of the values of these limits, at 1 bar and 25 °C the value of the reference diameter for air bubbles in water is 2.71 mm and the viscosity number is 2.02 × 10^−3^.

## 3. Obtaining the Flow Magnitudes from the Sensor Signals

One important question when using two and four sensor conductivity probes is how to obtain the values of the physical magnitudes of the flow from the sensor signals. In this section, a brief explanation of the methods we have used is given, and also in this section some references that we consider relevant are given for each method.

### 3.1. The Multi-Sensor Conductivity Probe as a Phase Identifier

The most obvious application of the two and four sensor conductivity probes is as phase identifiers. This application is obvious because the voltage signal provided by each sensor was different when the liquid phase or the gas phase touched any one of the conductivity probe tips. Therefore if we focused our attention on the signal provided by one of the tips, for instance the reference one, or any other one, as it was observed in Figure 7b that the electrical tension signal had sharp rises and falls. This allowed to identify the time instant at which a given bubble touched for the first time this conductivity sensor and therefore the gas phase was in contact with the tip, and the time instant at which this same bubble left the sensor. Because both time instants were recorded, then we could compute for this bubble the time interval $\Delta t_{g,i}$ that the gas phase of this bubble was in contact with the sensor. Therefore, the value of the average void fraction $\alpha (\vec{r})$ at the point where the tip was located is given by the well-known expression:

(7) $$\alpha (\vec{r})=\frac{\sum_{i=1}^{n}\Delta t_{g,i}}{T}$$

where *T* is the total sampling time and *n* the recorded number of bubbles during the sampling time. To calculate the void fraction we used the signal from the upstream tip. Now expression (7) needs a more detailed explanation, as in our case, due to the introduction of the threshold voltage, we can have an underestimation of the bubble residence time if we use the residence time from the binarized signal. We performed a correction of the apparent residence time of the bubbles to obtain the real one that is the sum of the apparent residence time of every bubble obtained from the binarized signal, $\Delta t_{ag,i}$, plus a small average correction denoted by ${\bar{\Delta t}}_{corrg}$, divided by the total measuring time [23,24]:

(8) $$\alpha =\frac{\sum_{i}\left(\Delta t_{ag,i}+{\bar{\Delta t}}_{corrg}\right)}{T}$$

**Figure 14:**
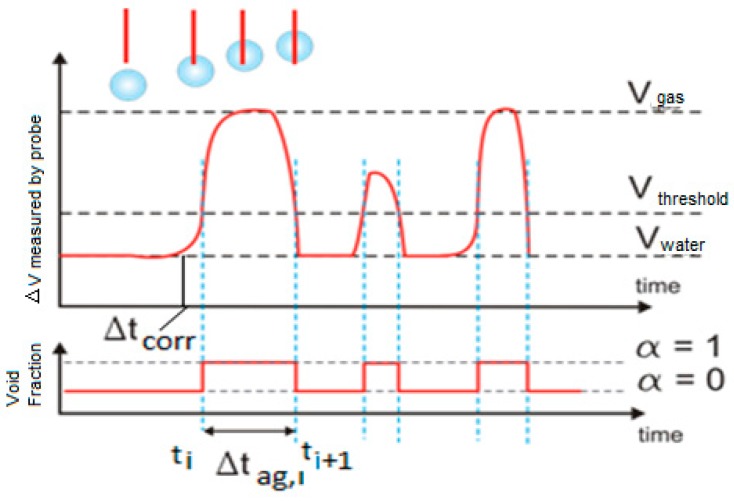
Scheme showing the correction of the bubble apparent residence time due to the threshold voltage.

To calculate the correction ${\bar{\Delta t}}_{corrg}$ to the apparent residence time, first we obtained the average of the voltage slope ${dV/dt|}_{threshold}$ at the point where the rising voltage signal cut the threshold voltage line as displayed at Figure 14. Then, the correction is given by:

(9) $${\bar{\Delta t}}_{corrg}=\frac{V_{threshold}}{{\frac{dV}{dt}|}_{threshold}}$$

### 3.2. Obtaining the Gas Velocity from the Signals Provided by the Sensors (Tips)

**Figure 15:**
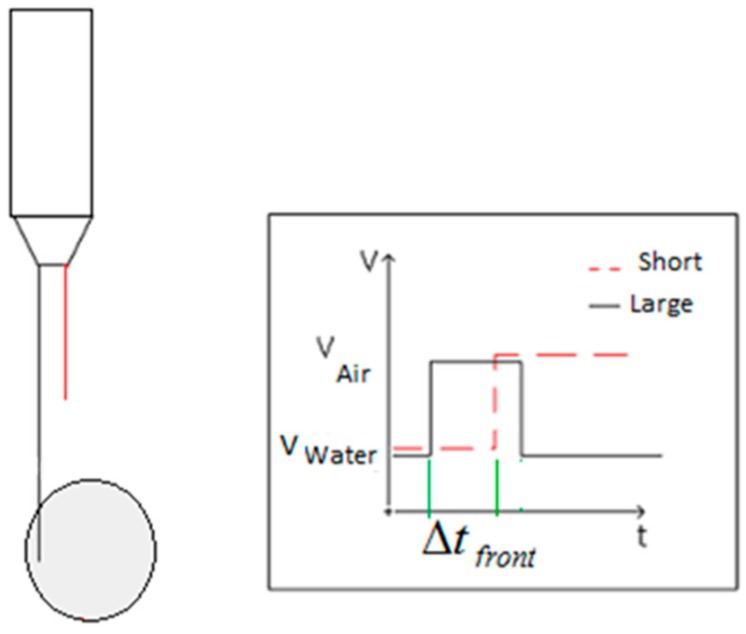
Time of flight $\Delta t_{front}$ measured using the bubble front interface and using the signals from the large and short tips.

Another application of the conductivity probe is the determination of the gas velocity from the signals provided by the tips. If we use the two tip conductivity probe then because we know the distance between the two tips, denoted by *S*, the component of the bubble interface velocity on the vector direction that goes from the larger to the shorter tip is given by:

(10) $$V_{m}=\frac{S}{\Delta t_{front}}$$

where $\Delta t_{front}=t_{S}-t_{L}$ is the time difference between the instant in which the bubble front touch the shorter tip $t_{S}$ and the instant in which the front touch the larger tip $t_{L}$ as displayed at Figure 15. Therefore, the average velocity ${\bar{V}}_{m}$ for a set of measurements performed with *n* bubbles, and using the front interface to measure the velocity, is given by:

(11) $${\bar{V}}_{m}=\frac{S}{n}\sum_{i=1}^{n}\frac{1}{\Delta t_{front,i}}$$

It is necessary to remark that when measuring the velocity we must use the signals produced by the front interface because the conductivity probe is an intrusive method and the tips could slow down the bubble velocity, as has been pointed out by Le Corre and Ishii [25].

The four tip conductivity probe provides more information than the two tip one. Shen et al. [18] developed methods to obtain the information from the four sensor signals. Denoting the position of the larger sensor tip by ${\vec{r}}_{0}$ and the position of the shorter tips by ${\vec{r}}_{k}$ *k* = 1, 2, 3. One can obtain easily the vectors with origin in the large tip and extreme in one of the shorter tips that are denoted by ${\vec{S}}_{0-k}={\vec{r}}_{k}-{\vec{r}}_{0}$. Because we can measure the time that need the interfacial front of any *j*-th bubble to travel from ${\vec{r}}_{0}$ to ${\vec{r}}_{k}$, then one can obtain the measured velocities of this *j*-th interface when traveling from the larger tip 0 to any one *k* of the shorter tips and that it is denoted by:

(12) $${\vec{V}}_{m,0-k,j}=\frac{{\vec{S}}_{0-k}}{t_{k,j}-t_{0,j}},\;k=1,2,3$$

**Figure 16:**
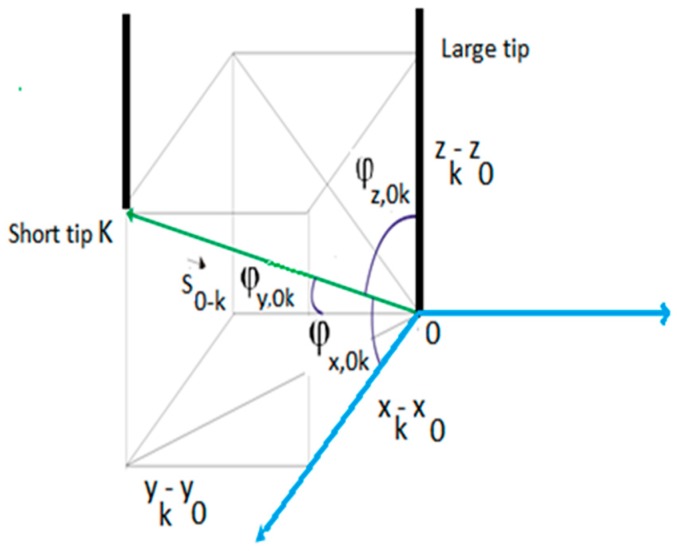
Positions and angles of the front and one of the rear sensors in Cartesian coordinates.

Denoting by ${\vec{n}}_{Ij}\left({\vec{r}}_{0}\right)$ the unit vector normal to the *j*-th interface at the point ${\vec{r}}_{0}$ where the larger tip touches the interface at the corresponding time. This unit vector in a Cartesian coordinates system will have three components that expressed in terms of its direction cosines yields:

(13) $${\vec{n}}_{IJ}\left({\vec{r}}_{0}\right)=\cos\phi_{x,Ij}\;{\vec{e}}_{x}+\cos\phi_{y,Ij}\;{\vec{e}}_{y}+\cos\phi_{z,Ij}\;{\vec{e}}_{z}$$

where $\left({\vec{e}}_{x},\;{\vec{e}}_{y},\;{\vec{e}}_{z}\right)$ denote the three unit Cartesian vectors. In addition, we can express the vector ${\vec{S}}_{0-k}$ in terms of its direction cosines (see Figure 16) as follows:

(14) $${\vec{S}}_{0-k}=\left|{\vec{r}}_{k}-{\vec{r}}_{0}\right|\left(\cos\phi_{x,0k}\;{\vec{e}}_{x}+\cos\phi_{y,0k}\;{\vec{e}}_{y}+\cos\phi_{z,0k}\;{\vec{e}}_{z}\right)\;k=1,2,3$$

The distances $\left|{\vec{S}}_{0-k}\right|$ for *k* = 1, 2, 3 are geometric distances, that are characteristics of each particular four sensor conductivity probe, and are measured during the building process, also the direction cosines $\left(\cos\phi_{x,0k},\;\cos\phi_{y,0k},\;\cos\phi_{z,0k}\right)$ depend on the geometric characteristics of the conductivity probe and therefore are previously known magnitudes. Then for each sensor *k* = 1, 2, 3, and each bubble front *j* we can measure its interfacial velocity from the large tip to the shorter tip *k* = 1, 2, 3:

(15) $${\vec{V}}_{m,0-k,j}=\frac{\left|{\vec{r}}_{k}-{\vec{r}}_{0}\right|}{t_{k,j}-t_{0,j}}\left(\cos\phi_{x,0k}\;{\vec{e}}_{x}+\cos\phi_{y,0k}\;{\vec{e}}_{y}+\cos\phi_{z,0k}\;{\vec{e}}_{z}\right)\;k=1,\;2,\;3$$

The next step is to use the Interfacial Measurement Theorem for multi-sensor conductivity probes obtained by Shen et al. [18]. This theorem provides the relationship between the unknown local interfacial velocity and the measured interfacial velocities ${\vec{V}}_{m,0-k,j}$ for any multi-sensor conductivity probe with an arbitrary number of tips. If we denote by ${\vec{V}}_{I,j}$ the velocity of the *j*-th interface at the sensor point ${\vec{r}}_{0}$ and at the time instant $t_{0,j}$ that this interface touches the large tip, then this theorem says that the following identity can be established [18]:

(16) $$V_{Ij,n}={\vec{n}}_{Ij}\cdot {\vec{V}}_{I,j}={\vec{n}}_{ij}\cdot {\vec{V}}_{m,0-k,j}$$

where ${\vec{n}}_{Ij}={\vec{n}}_{ij}\left({\vec{r}}_{0}\right)$ denotes the unit vector normal to the *j*-th interface at the point ${\vec{r}}_{0}$ that is usually written omitting ${\vec{r}}_{0}$ and $V_{Ij,n}$ denotes the normal component of the interfacial velocity of the *j*-th front. We observe that Equation (16), provides for the four sensor conductivity probe a linear systems of 3 equations with four unknowns that are $\cos\phi_{x,Ij}$, $\cos\phi_{y,Ij}$ $\cos\phi_{z,Ij}$, and $V_{Ij,n}$ but the summation of the squares of the director cosines is unity so really we have only three unknowns:

(17) $$\left(\cos\phi_{x,Ij}\;\cos\phi_{x,0k}+\cos\phi_{y,Ij}\;\cos\phi_{y,0k}+\cos\phi_{z,Ij}\;\cos\phi_{z,0k}\right)=V_{Ij,n}\frac{t_{k,j}-t_{0,j}}{s_{0-k}}\;k=1,2,3$$

Solving the linear equation system (17) by the Cramer rule and using the well-known relation ${\left(\cos\phi_{x,Ij}\right)}^{2}+{\left(\cos\phi_{y,Ij}\right)}^{2}+{\left(\cos\phi_{z,Ij}\right)}^{2}=1$ yields for an oncoming surface moving upward:

(18) $$\cos\phi_{x,Ij}=\frac{V_{Ij,n}\cdot A_{1,j}}{A_{0}},\;\cos\phi_{y,Ij}=\frac{V_{Ij,n}\cdot A_{2,j}}{A_{0}},\;\cos\phi_{z,Ij}=\frac{V_{Ij,n}\cdot A_{3,j}}{A_{0}}$$

and for the normal component of the interfacial velocity, i.e., the interfacial velocity projected onto the normal to the bubble front:

(19) $$V_{Ij,n}=\frac{A_{0}}{\sqrt{A_{1,j}^{2}+A_{2,j}^{2}+A_{3,j}^{2}}}$$

where the determinants $A_{0},\;A_{1,j},\;A_{2,j},\;A_{3,j}$ coming from the Cramer’s solution are given by:

(20) $$A_{0}=\left|\begin{array}{ccc} \cos\phi_{x,01} & \cos\phi_{y,01} & \cos\phi_{z,01}\\ \cos\phi_{x,02} & \cos\phi_{y,02} & \cos\phi_{z,02}\\ \cos\phi_{x,03} & \cos\phi_{y,03} & \cos\phi_{z,03} \end{array}\right|$$

The determinants $A_{1,j},\;A_{2,j},\;A_{3,j}$ are obtained substituting the first, second, and third column respectively of the determinant $A_{0}$ by the column vector: $column\left(\frac{t_{1,j}-t_{0,j}}{s_{0-1}},\;\frac{t_{2,j}-t_{0,j}}{s_{0-2}},\;\frac{t_{3,j}-t_{0,j}}{s_{0-3}}\right)$.

Finally, the Cartesian components of the projection of the interfacial bubble’s velocity onto the normal to the interfacial area are:

(21) $${\vec{V}}_{Ij,n}=V_{Ij,n}\;{\vec{n}}_{I,j}=V_{n,j}(\cos\phi_{x,Ij}\;{\vec{e}}_{x}+\cos\phi_{y,Ij}\;{\vec{e}}_{y}+\cos\phi_{z,Ij}\;{\vec{e}}_{z})$$

### 3.3. Calibration Factors for the Bubble Velocity

The interfacial velocity obtained from the two sensor conductivity probe, and given by Equation (11) is not the true interfacial velocity, and in order to obtain the true velocity one must multiply the experimental result by the so called “velocity calibration factor”. The velocity calibration factors $f_{v}$ are defined as the ratio of the mean velocity of the bubbles $\bar{V}$ and the mean measured velocity ${\bar{V}}_{m}$ [25,26,27]:

(22) $$f_{v}=\frac{\bar{V}}{{\bar{V}}_{m}}$$

In practice is very complex to relate the average measured velocity or interfacial velocity of the bubbles denoted by ${\bar{V}}_{m}$ with the true mean velocity of the bubbles $\bar{V}$. The measured velocity with the two sensors conductivity probe depends of several factors, as pointed out by Le Corre and Ishii [25]. These factors depend on the sensor response, because one can never measure the exact time instant in which the probe touch the bubble as explained previously because during the binarization process this time value can have small errors, and also have the bubble velocity fluctuations that influence the result. In addition, the geometry of the sensor and the size and shape of the bubbles also influence the results. For this reason, Wu et al. [26], Le Corre and Ishii [25], Muñoz-Cobo et al. [27] developed in the past several methods to compute this velocity calibration factor.

### 3.4. Roots of the Method to Measure the Interfacial Area Concentration by Means of Multi-Sensor-Conductivity Probe

The roots of the method to measure the interfacial area concentration were established by Delhaye [28], and Delhaye and Achard [29]. If we have a control volume *V*, and inside this volume we have a biphasic flow and we denote by $A_{I}\left(t\right)$, the total interfacial area or area of all the moving boundaries contained in *V* at time *t*, and by $a_{I}\left(t\right)$ the instantaneous interfacial area concentration inside the volume *V* at time *t*, i.e., $a_{I}\left(t\right)=A_{I}\left(t\right)/V$. Then, Delhaye proved rigorously that for any continuous vector field ${\vec{B}}_{k}\left(\vec{r},\;t\right)$ in the *k*-th phase (liquid or gas), the following identity holds:

(23) $$\int_{V}\sum_{j}\frac{{\left({\vec{B}}_{k}(\vec{r},t)\cdot {\vec{n}}_{k}\right)}_{j}}{l_{j}}dV=\bar{\int_{A_{I}(t)}{\vec{B}}_{k}(\vec{r},t)\cdot {\vec{n}}_{k}\;dA}$$

where $l_{j}=T{\left|{\vec{v}}_{I}\cdot {\vec{n}}_{k}\right|}_{j}$ is the distance travelled by the *j*-th moving interface in the direction ${\vec{n}}_{k}$ of the unit vector normal to this interface of the *k*-th phase, the summation *j* extends over all the moving interfaces passing through $\vec{r}$ during the interval *T*. The average symbol in the right hand side for any magnitude $g(\vec{r},t)$ must be understood as follows:

(24) $$\bar{\left(g\right)}=\frac{1}{T}\int_{\left[T\right]}g(\vec{r},t)dt$$

where the symbol [T] denotes a time interval centered at *t*, i.e., $\left[t-\frac{T}{2},t+\frac{T}{2}\right]$. Selecting the field as ${\vec{B}}_{k}={\vec{n}}_{k}$ on the interfaces, then Equation (23) simplifies to the expression:

(25) $$\int_{V}\sum_{j}\frac{1}{l_{j}}dV=\bar{A_{I}(t)}\Rightarrow \frac{1}{V}\int_{V}\sum_{j}\frac{1}{T{\left({\vec{v}}_{I}\cdot {\vec{n}}_{k}\right)}_{j}}dV=\bar{\left(\frac{A_{I}(t)}{V}\right)}=\bar{a_{I}(t)}$$

Finally, from expression (25) it is obtained the expression for the local interfacial area concentration that must be measured with the conductivity probes:

(26) $$a_{I}(\vec{r},t)=\frac{1}{T}\sum_{j}\frac{1}{{\left|{\vec{v}}_{I}\cdot {\vec{n}}_{k}\right|}_{j}}$$

where the summation extends over all the interfaces passing thought the point $\vec{r}$, during the time interval [*t-T*/2, *t+T*/2].

### 3.5. Obtaining the Interfacial Area Concentration from the Signals Provided by the Multi-Sensor Conductivity Probe

If *S* denotes the distance between the front sensor (larger one), and the rear sensor (shorter one) in the two sensor conductivity probe and by $\theta_{j}$ the angle between the normal to the bubble interface at the tip hitting point for the *j*-th bubble front and the bubble velocity ${\vec{v}}_{I}$, then we can recast expression (26) as follows:

(27) $${\bar{a}}_{I}(\vec{r})=\frac{1}{T}\sum_{j=1}^{2N_{b}}\frac{1}{{\left|{\vec{v}}_{I}\cdot {\vec{n}}_{I}\right|}_{j}}=\frac{1}{T}\sum_{j=1}^{2N_{b}}\frac{\Delta t_{front,j}}{S\;\cos\theta_{j}}=\frac{2N_{b}}{T}\bar{\left(\frac{\Delta t_{front}}{S\cos\theta }\right)}$$

where the overbar means the averaging operation performed over the measured bubbles interfaces. We have assumed that during the counting interval *T* will pass $2N_{b}$ interfaces trough the conductivity probe sensors. Then assuming that the angle $\theta_{j}$ and the interfacial velocity are uncorrelated, Equation (27) can be expressed as follows [14,16]:

(28) $${\bar{a}}_{I}(\vec{r})=\frac{2N_{b}}{T}\bar{\left(\frac{\Delta t_{front}}{S}\right)}\int_{0}^{\pi /2}\frac{P(\theta )}{\cos\theta }d\theta =4f_{b}\bar{\left(\frac{1}{V_{m}}\right)}$$

where $f_{b}$ is the number of bubbles passing though the sensor tip per unit time. The average value of 1/cosθ, can be replaced by an integral over its probability density function P(θ), that yields a value of 2. So that expression (28) can be used to compute the interfacial area concentration in the two sensor conductivity probe.

For the four sensors conductivity probe, because of Equations (19) and (26) it is obtained that the interfacial area concentration is given by the expression:

(29) $${\bar{a}}_{I}(\vec{r})=\frac{1}{T}\sum_{j}\frac{1}{{\left|{\vec{v}}_{I}\cdot {\vec{n}}_{k}\right|}_{j}}=\frac{1}{T}\sum_{j}\frac{\sqrt{A_{1,j}^{2}+A_{2,j}^{2}+A_{3,j}^{2}}}{A_{0}}$$

Therefore, Equations (27) and (29) give the formulas to obtain from the signals the interfacial area concentration for the two and four tip conductivity probes, respectively. The interfacial area concentration (IAC) obtained from Equations (27) or (29) must be multiplied by the calibration factor for IAC denoted $f_{{\bar{a}}_{I}}$ [15,27].

### 3.6. Measurement of the Liquid and Gas Superficial Velocities

The liquid flow rate (m^3^/s) was obtained from the void fraction and the liquid velocity at each radial position performing the following integral:

(30) $$Q_{l}=\int_{0}^{R}v_{l}(r,z)(1-\alpha (r,z))2\pi dr\approx \sum_{i=1}^{15}v_{l}(r_{i},z)(1-\alpha (r_{i},z))2\pi r_{i}\Delta r_{i}$$

The liquid velocity was measured by using the LDA equipment described earlier, while the void fraction was measured with the sensor conductivity probe. Then, the superficial velocity was obtained by means of the expression $j_{l}=Q_{l}/A$. In addition, the superficial velocity was obtained from the flow provided by the electromagnetic flowmeter divided by the area of the pipe i.e., $j_{l,emf}=Q_{l,emf}/A$. Table 3 displays the results obtained by both methods.

To obtain the gas superficial velocity $j_{g}$ we measured, at a given height, the void fraction and the interfacial velocity at 15 radial positions $r_{i}$. Then we calculated the volumetric flow rate $Q_{g}$ from the following expression:

(31) $$Q_{g}=\int_{0}^{R}v_{g}(r,z)\alpha (r,z)2\pi dr\approx \sum_{i=1}^{15}v_{I}(r_{i},z)\alpha (r_{i},z)2\pi r_{i}\Delta r_{i}$$

The gas superficial velocity was obtained from the expression $j_{g}=Q_{g}/A$. To compute the measurement error for instance for the superficial velocity obtained with the electromagnetic Rosemount flow meter that have a standard accuracy of 0.5%, we used the expression:

(32) $$\varepsilon (j_{l,emf})=\left|\partial j_{l,emf}/\partial Q_{l,emf}\right|\varepsilon (Q_{l,emf})+\left|\partial j_{l,emf}/\partial A\right|\varepsilon (A)$$

The relative error is after some calculus given by:

(33) $$\varepsilon (j_{l,emf})/j_{l,emf}=\varepsilon (Q_{l,emf})/Q_{l,emf}+\varepsilon (A)/A=0.5\times 10^{-2}+0.30\times 10^{-2}=0.8\times 10^{-2}$$

Therefore, we have an accuracy of 0.8% for the liquid superficial velocity measured with the electromagnetic gauge.

### 3.7. Experimental Results

We selected the flow characteristics to cover a wide range of liquid and gas flow conditions ranging from bubbly to the transition to cap/slug flow. Then, we measured the superficial liquid and air velocities and the average void fraction at z/D = 56 for each flow condition with the conductivity probes F0X and F0A. Most of the conditions displayed at Table 4 and Table 5 are in the bubbly flow regime, and only for the highest void fraction values some large cups appear, near the transition. For each liquid velocity conditions we have at least five gas conditions with void fractions ranging from 5% to up 25%, except for the case $j_{f}$ = 0.5 m/s where an extra case was added near the transition from bubbly to cap/slug regime. At the setup of each run the void fraction was measured by a pressure sensor located at z/D = 56.

The experimental flow boundary conditions for the experiments performed with the sensors F0X and F0A were very close except for experiments F0A1G06 and F0X1G06. In this case, the gas superficial velocity $j_{g}$ for the experiment performed with the sensor F0A was an 18% higher than for the experiment performed with the sensor F0X.

It is important to understand the nomenclature used to denote the experiments. As an example, for the case F0A1G05, the first three characters denote the sensor used in the experiment, the following character ranging from 1 to 4 denotes the liquid superficial velocity boundary condition where 1 is equivalent to (0.5 m/s), 2 to (1 m/s), 3 to (2 m/s), 4 to (3 m/s). Then the symbol G denotes the gas boundary condition and 00 means that we have not gas, then the next two characters ranging from 01, to 06 means increasing gas superficial velocities with increasing void fraction values.

**Figure 17:**
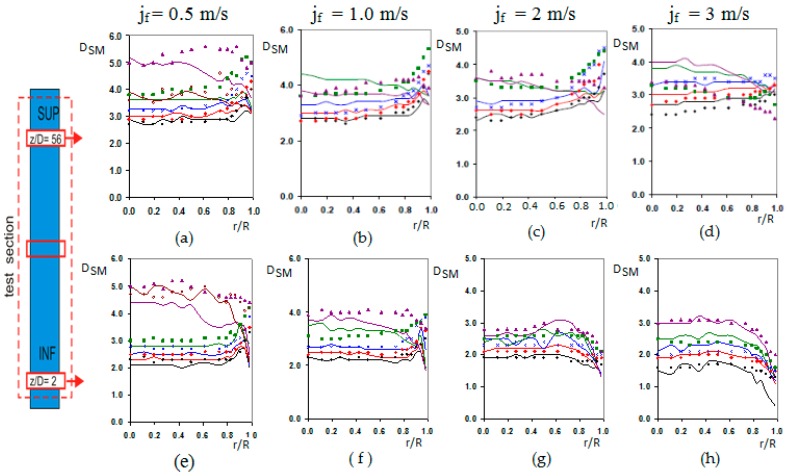
Profiles of the Sauter Mean Diameter D_SM_ [mm] versus the radial position (r/R). Solid symbols correspond to F0A probe, and without symbol (continuous line) to F0X probe. The legend for the graphs is as follows: Black (●) G01, Red (◊) G02, Blue (X) G03, Green (■) G04, Violet (▲) G05, light Violet (○) G06. (**a**–**d**) measurements performed at the upper port for $j_{f}=0.5,\;1,\;2,\;3\;m/s$. (**e**–**h**) measurements performed at the lower port for $j_{f}=0.5,\;1,\;2,\;3\;m/s$.

The bubble diameter at the test section inlet depends on the gas flow, the liquid flow, and the bubble generator system, for given conditions of pressure. With liquid velocities at the inlet below 2 m/s the turbulence does not produce substantial bubble disintegration, and the initial bubble size determinates the bubble diameters and void fraction profiles. The bubble diameter at the inlet was in the range [2,3,4] mm, as observed in Figure 17b–h, and only for $j_{f}$ = 0.5, G05 ($<\alpha_{sup}>\approx$ 21%) and G06 ($<\alpha_{sup}>\approx$ 28%), Figure 17a,e larger diameters were detected. Then, the bubble diameter increases its value mainly by the pressure reduction along the flow direction as observed comparing Figure 17a, upper port, and Figure 17e, lower port. We observed that the bubble Sauter mean diameter is bigger in the upper port than in the lower port for the same case.

**Figure 18:**
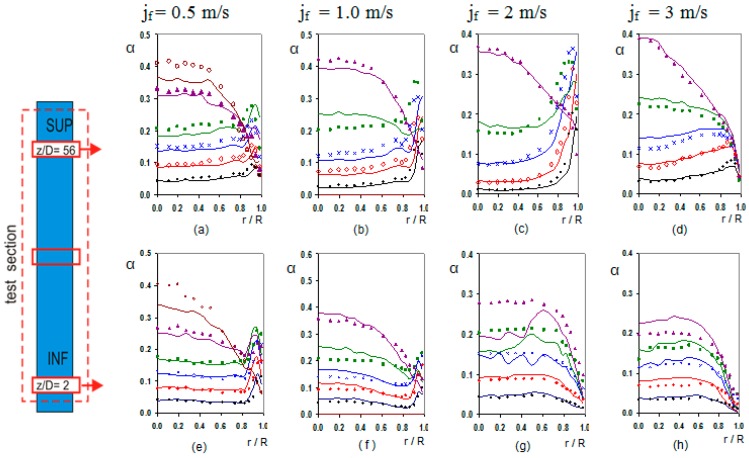
Void fraction average profile [-] vs. the radial position (r/R). The solid symbols correspond to F0A probe, and without symbol (continuous lines) to F0X probe. The legend for the graphs is as follows: Black (●) G01, Red (◊) G02, Blue (X) G03, Green (■) G04, Violet (▲) G05, light Violet (○) G06. (**a**–**d**) measurements performed at the upper port for $j_{f}=0.5,\;1,\;2,\;3\;m/s$. (**e**–**h**) measurements performed at the lower port for $j_{f}=0.5,\;1,\;2,\;3\;m/s$.

The radial distribution of the bubble, gas phase, depends on the bubble diameter, and the liquid velocity gradient. Small bubbles tend to move toward the wall, while large bubbles migrate preferably to the centre. This lateral motion is determined by the lift force and its sign changes as a function of the bubble diameter [11,12]. In addition, we must consider another force, the wall lubrication force, which drives the bubble away from the wall, but its effect decreases very fast with the distance to the wall beyond some millimetres. The balance, between these two forces, produces a peak near the wall for the concentration of small bubbles [13]. Figure 18 displays the void fraction distribution with the radial distance to the tube centre for several cases. We can observe the three typical profiles, wall-peak, transitional and core-peak. We notice that for $j_{f}$ = [0.5–2] m/s the void fraction distribution show a wall-peak profile for G01,G02 and G03, cases typically located in the void fraction range [0–15%]. The local void fraction profile has the typical evolution with the pressure reduction, and the consequent increase in bubble size. The peaks in the void fraction profile were located more or less at one bubble diameter from the wall. For $j_{f}$ = 2 m/s and 3 m/s at the lower position, in Figure 18g,h no wall-peak profile was observed for any void fraction, perhaps, the development zone at the entrance produces a bubble migration toward the centre, since the bubble diameter is small. For G04 ($<\alpha_{sup}>\approx$ 20%), the profile has an evolution to transitional shape, due to the tendency of the large bubbles to migrate toward the channel centre, and only shows wall-peak for $j_{f}$ = 0.5 m/s at z/D = 2, Figure 18e. For $j_{f}$ = 2 m/s and 3 m/s at z/D = 2 the void fraction has a transitional profile for all the void fractions. The core-peak profile appears when a large quantity of gas is injected to the system in this case large bubbles appear migrating towards the centre of the tube, as observed in Figure 18a–e for G05 and G06 cases. For average void fraction larger than 20% a core-peak profile appears, mainly in the upper part of the test section. Figure 17a,e show the presence of large bubbles for low $j_{f}$. For $j_{f}$ larger than 2 m/s cups were detected, but their diameter is not shown in Figure 17, as only bubbly flow was studied.

The interfacial gas velocity has a flat profile for cases with wall-peak, when the average void fraction is lower than 20%. This matches the flat void fraction profile far from the wall where the peak appears. Near to the wall, the interfacial velocity decreases due to the wall influence and the drag force produced by the liquid. With a liquid superficial velocity less than 2 m/s and wall-peak void fraction profiles, the increase of $j_{g}$ has little influence on the gas velocity profile. We observed that the gas velocity increased substantially when large bubbles appeared in the flow, due mainly to the buoyancy force.

### 3.8. Validation of the Measurements of Two-Phase Flow Parameters Using the Conductivity Probes

**Figure 19:**
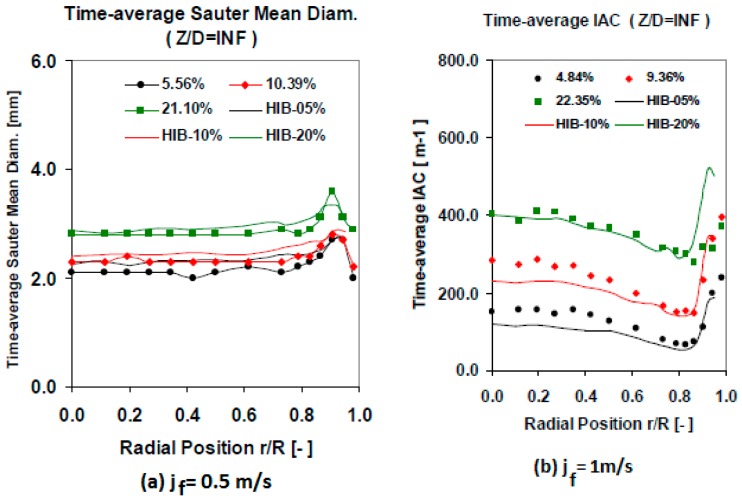
Comparison with the values obtained by Hibiki [30,31]: (**a**) Sauter mean diameter [m] vs. the radial position (r/R), for different void fraction values with $j_{f}$ = 0.5 m/s. (**b**) Interfacial area concentration [m^−1^] vs. the radial position (r/R) for different void fraction values with $j_{f}$ = 1 m/s, measured with the F0X four tips probe.

**Figure 20:**
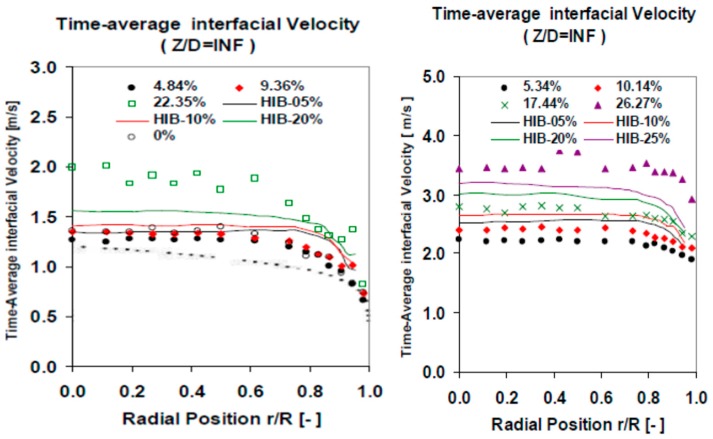
Comparison with the values obtained by Hibiki [30,31] for the time averaged interfacial velocity. The measures were performed with the F0X sensor and $j_{f}=1.038\;m/s$ (**left**), and with the F0A sensor and $j_{f}=2.042\;m/s$ (**right**), at several gas flow conditions and at the lower port.

A set of cross checking measurements were performed to validate the experimental results obtained using the multi-sensor conductivity probe. The cross checking was performed comparing the conductivity sensor results with other measurement methods and with the results of other authors using the same flow boundary conditions and geometry. First, we performed several measurements using flow conditions close to the ones used by Hibiki et al. [30,31]. The results obtained for the Sauter mean diameter were similar to the ones obtained by Hibiki, for different void fraction values and with $j_{f}$ = 0.5 m/s, as displayed in Figure 19a. In addition, the results for the interfacial area concentration (IAC) at the lower port, were very similar to the ones obtained by Hibiki, as displayed in Figure 19b. This comparison was performed for different void fraction values with $j_{f}$ = 1 m/s. We observed that for the biggest void fraction value our F0X sensor measured a smaller peak near the wall than in Hibiki case, this was produced because in our case the void fraction was 22.35% while in Hibiki case was 20%, and for this case start the transition from wall peak to core peak. Finally at Figure 20 we compared the values obtained with $j_{f}$ = 1.038 m/s (left) and $j_{f}$ = 2.042 m/s (right) for the interfacial velocity at different gas flow conditions. The differences are within 10% for all the cases, except for the highest void fraction, however in this case our void fraction was 10% bigger than Hibiki one, so this can produce some differences.

In addition, we measured also the pressures with a pressure sensor. The values provided by the pressure gauge depend on the water column above this sensor and the pressure drops of the fluid flowing through the pipe. Because of Figure 8, we can relate the pressures provided by the pressure gauge for the single and the two-phase flow cases with the weight of the columns above the gauge and the friction drops as follows:

(34) $$p_{abs,1\phi }=p_{atm}+\rho_{l}g\;h+\Delta p_{fric,1\phi }$$

(35) $$p_{abs,2\phi }=p_{atm}+\rho_{m}g\;h+\Delta p_{fric,2\phi }$$

where $p_{abs,1\phi }$, and $p_{abs,2\phi }$ are the absolute pressures for the single and the two phase flow cases; $p_{atm}$ is the atmospheric pressure; $\Delta p_{fric,1\phi }$, and $\Delta p_{fric,2\phi }$ are the pressure drops for the single and two phase flow cases; $\rho_{l}gh$ and $\rho_{m}g\;h$ are the water column pressures for the single phase and the two phase cases respectively. Finally $\rho_{m}=\left(1-\alpha \right)\rho_{l}+\alpha \rho_{g}$ is the density of the biphasic mixture. Subtracting equations (34) and (35), and because of the approximation $\rho_{l}-\rho_{m}=\alpha \left(\rho_{l}-\rho_{g}\right)\approx \alpha \rho_{l}$, yields the following expression for the void fraction:

(36) $$\alpha =\frac{p_{rel,1\phi }-p_{rel,2\phi }+\Delta p_{fric,1\phi }-\Delta p_{fric,2\phi }}{p_{rel,1\phi }-\Delta p_{fric,1\phi }}$$

where $p_{rel,1\phi }=p_{abs,1\phi }-p_{atm}$ and $p_{rel,2\phi }=p_{abs,2\phi }-p_{atm}$ are the relative pressures with respect to the atmospheric pressure for the single-phase and the two-phase cases respectively. To obtain the two-phase pressure drop we use the following standard expression [32]:

(37) $$\Delta p_{fric,2\phi }=f_{l0\;}\phi_{l0}^{2}\left(\frac{\Delta Z}{D_{h}}\right)\left(\frac{G_{T}^{2}}{2\rho_{l}}\right)$$

where $f_{l0}$ is the friction factor that is related to the Reynolds number by the Blasius expression $f_{l0}=0.316\;Re^{-0.25}$; $G_{T}=\rho_{g}j_{g}+\rho_{l}j_{l}$ is the total mass flow rate; $D_{h}$ is the hydraulic diameter and $\phi_{l0}^{2}$ is the well-known two-phase frictional multiplier [32]:

(38) $$\phi_{l0}^{2}=\left(1+x\frac{\rho_{l}-\rho_{g}}{\rho_{g}}\right){\left(1+x\frac{\mu_{l}-\mu_{g}}{\mu_{g}}\right)}^{-0.25}$$

Being x the dynamic quality obtained from the expression $x=\left(\rho_{g}j_{g}\right)/(\rho_{g}j_{g}+\rho_{l}j_{l}$). The single-phase pressure drop was obtained using the same method but with $\phi_{l0}^{2}$ equal to 1, on account that the dynamic quality for this case was zero. Then we compared the average void fractions obtained by the conductivity probe with the void fractions obtained from the pressure sensors for the experiments F01, F02, F03, F04, F0A1, F0A2, F0A3 and F0A4. The boundary conditions, i.e., the superficial velocities for the gas and liquid phases, for these experiments are displayed in Table 4 and Table 5. For void fractions smaller than 25%, the differences observed between the void fractions obtained with both methods were always smaller than 10% or close to this error band for practically all the cases, in all the runs F0X1 to F0X4, and F0A1 to F0A4.

**Figure 21:**
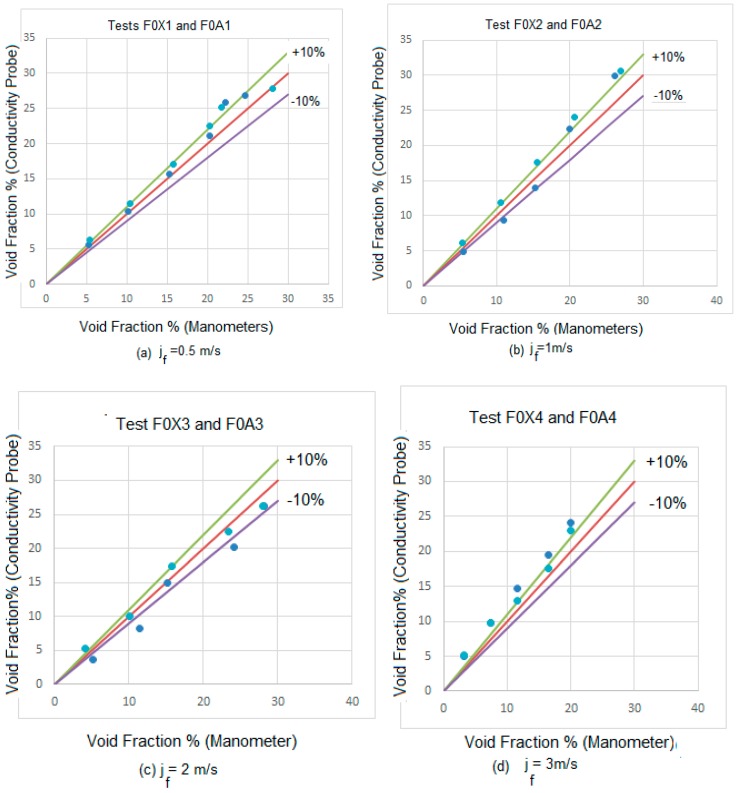
Experimental void fraction obtained from the manometer measurements (*x*-axis), and from the conductivity sensor (*y*-axis), at the upper port for different gas flow and liquid flow conditions.

In Figure 21a–d we display the results obtained for the void fraction using both conductivity probes and manometers at liquid superficial velocities of $j_{f}$ = 0.5 m/s, 1 m/s, 2 m/s and 3 m/s. We observed that for the lower void fractions both methods provide the same results in a ±10% band (continuous violet and green lines). However, for void fractions above 20% some differences appear for some cases that are due to some large caps that produce oscillations in the liquid phase inducing errors in the pressure gauges. However these differences are not important because even at high void fraction most of the point are within the ±10% band or close to this band as observed in Figure 21a–d.

**Figure 22:**
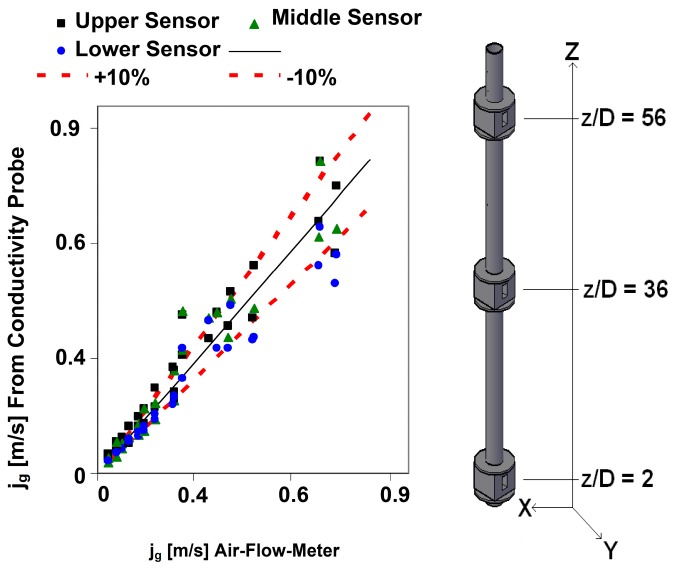
Experimental gas superficial velocities at three axial positions (Upper, Middle, and Lower), measured with the sensor conductivity probe and with a gas flow meter at UPV in PUMA facility.

In addition, we have compared the volumetric gas flow rate injected in the water column and measured with an air flow meter with the volumetric gas flow rate measured using the multi-sensor conductivity probe as explained in Section 3.6. We display in Figure 22 the gas superficial velocities $j_{g}=Q_{g}/A$ obtained using the air flow meter and the sensor conductivity probe, for a set of cases with liquid velocity 0.5 m/s when no gas was injected and void fractions ranging from 5 to 25%. The sensor conductivity probe was located at three heights in the channel *z/D* = 2, *z/D* = 36, and *z/D* = 56, displayed in Figure 22. Most of the data are within the band ±10%, as observed in Figure 22.

To finish this section we performed an evaluation of the uncertainty for some cases and magnitudes using standard methods of uncertainty propagation. The uncertainty in the superficial velocities of the gas was ±11% and for the liquid was ±8%. In this last case, we compared our results with the results obtained from an electromagnetic gauge with very small measurement error and the differences found confirmed our estimation of the uncertainty in the measurements. For the void fraction, we evaluated an uncertainty of ±10% that we confirmed by measurements performed with a manometer as discussed in the conclusions. To evaluate the uncertainty or dispersion of the data values attributed to a given measured magnitude as for instance the gas superficial velocity $j_{g}$ obtained from the conductivity probe measurements at 15 radial points. This magnitude, on account of Equation (31), is a function that can be expressed as $j_{g}=f(\alpha_{1},\alpha_{2},\ldots ,\alpha_{15},\;r_{1},r_{2},\ldots r_{15},A$). The arguments of this function are the void fraction values, the radial distances at the measurement points, and the pipe area. Then the uncertainty in the output is computed from the expression:

(39) $$\sigma (j_{g})=\sqrt{\sum_{i=1}^{15}{\left(\frac{\partial j_{g}}{\partial \alpha_{i}}\right)}^{2}\sigma^{2}(\alpha_{i})+\sum_{i=1}^{15}{\left(\frac{\partial j_{g}}{\partial r_{i}}\right)}^{2}\sigma^{2}(r_{i})+{\left(\frac{\partial j_{g}}{\partial A}\right)}^{2}\sigma^{2}(A)}$$

The values of $\sigma^{2}\left(\alpha_{i}\right)$ are estimated from their variances $s^{2}\left(\alpha_{i}\right)$.

## 4. Conductance Probes for Annular Flow

In this section, we describe briefly the conductance probes developed to study the interfacial waves produced in annular flow. Due to the low visibility that we have in the annular flow regime produced by the mist formation, inside the experimental channel, and the production of droplets in the crest of the disturbances waves. Then, we could not take good pictures with borescopes introduced inside the experimental channel, so we decided to use conductance probes to study the different class of interfacial waves produced in the annular flow regime.

### 4.1. Sensor Performance

The conductance sensor uses the water conductivity to obtain the thickness value of the liquid film [33,34]. It consists of three electrodes mounted flush to the wall, aligned in the same flow direction of the liquid film and gas flow. The electrode located in the center is connected to the ground. One of the side electrodes is excited by a 300 kHz and 4 V peak-to-peak sinusoidal signal, and the other one is the receiver electrode, which receives the electric current that the water conduces. The amount of current that the receiver electrode collects is proportional to the liquid film thickness. This is because there are more electric field lines in a thicker liquid film and therefore more electric current reaches the receiver sensor [33]. When an interfacial film wave crosses the sensor, an electric signal is obtained, which shows the thickness variation with time. The signal is registered on the data acquisition system after it is rectified and amplified by an electronic circuit that will be explained below. At the same time, this signal gives us the waveform when we plot it.

### 4.2. Sensor Design

**Figure 23:**
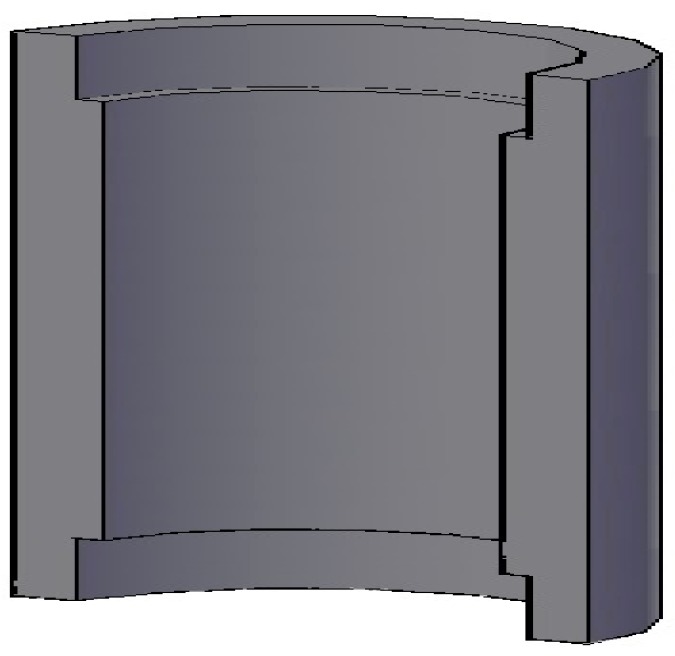
Schematic of the port for the conductance probes.

**Figure 24:**
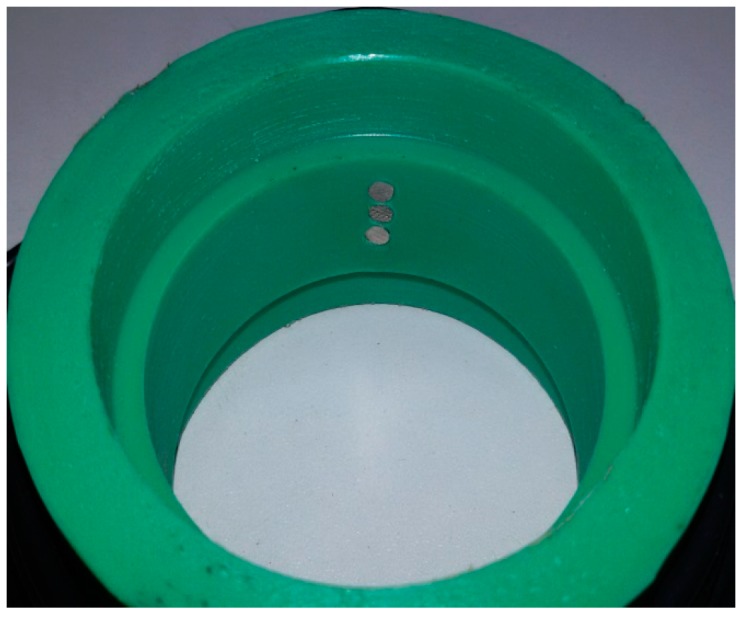
Location of the electrodes in the port.

To construct the device that permits to insert the sensor in the test section we have used a Poly-Propylene (PPR) pipe. This pipe can be machined easily. The length of the port is 15 mm and the internal diameter is the same as the measuring pipe. Slots have been performed at both ends so that the measuring tube fits perfectly and does not cause any disturbance in the liquid film (see Figure 23). The electrodes have been placed in the central part of the port (see Figure 24).

We have built two sensors. The first one consists of three electrodes with 2 mm of diameter separated 1.5 mm between them, and the second one has three electrodes with 3 mm of diameter separated 1.5 mm. We mounted both sensors in a pipe with 45 mm of internal diameter.

In this type of sensors, the distance between the electrodes and the electrode diameter is very important. Using a smaller separation, we obtained better resolution, but it is recommended that this distance be greater than the electrode diameter. In addition, a small electrode diameter can follow in more detail the shape of the waves. The waveforms recorded by both sensors are slightly different and the voltage values obtained by the second sensor are lower than the values obtained by the other one. All the cables used to connect the electrodes with the electronic part have been shielded. The connection of the electrodes with the cable in each port is different. In one of them, we have connected it by TIG welding, joining the copper with stainless steel using a stainless steel rod, while in the other probe we have used epoxy resin. In this way, we will be able to evaluate whether the welding affects the conductive properties of the electrodes, due to the heat of the welding and the quenching of the cooling, since the structure of the steel can be modified. A TTi TG315 signal generator (Texas Instruments, Dallas, TX, USA) has been used to generate the excitation signal. The reason why we use a sinusoidal signal is that it does not polarize the water. Since the signal has a positive and a negative component, we avoid corrosion in the electrodes; whereas with a signal in direct current, the electrodes would corrode.

**Figure 25:**
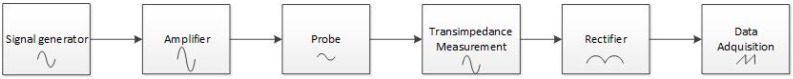
Flow diagram of the probe electronic circuit.

The sensors electronic circuit, displayed at Figure 25, amplifies and isolates the sinusoidal signal from the signal generator before sending it to the excitation electrode and captures the signal that the receptor electrode receives. The received signal passes through a trans-impedance measurement operational-amplifier configuration and it is buffered before the rectification stage, where AC signal is converted to a proportional DC voltage. Then, the data acquisition system acquires this DC signal, whose value is related with the liquid film thickness. Then, the variations in the value of the DC signal will indicate the passage and height of the waves present in the liquid film.

### 4.3. Sensor Calibration

In this section, we explain the method used to perform the sensor calibration. The objective was to relate the signal voltage with the water film height, in order to relate the information contained in the signals with the amplitude of the waves produced in the fluid interface. We used two types of conductance sensors, both types with three electrodes, and 2 mm of diameter for the electrodes of the first type, and 3 mm of diameter for the electrodes of the second type. These electrodes were equally spaced with 1.5 mm between them, aligned in the direction of flow and flush in the inner wall of the pipe.

**Figure 26:**
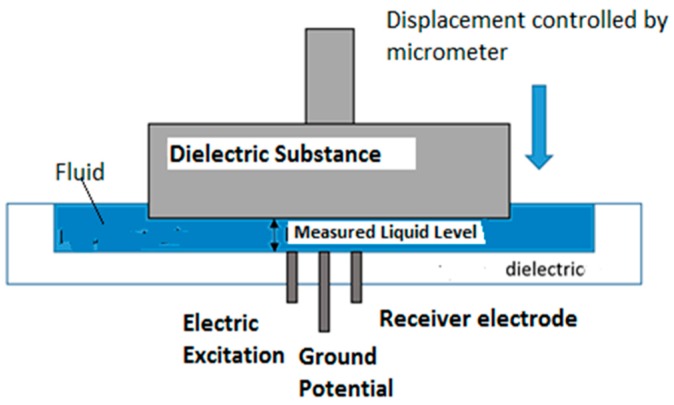
Assembly to calibrate the sensor.

To perform the calibration we used the following configuration displayed at Figure 26. In this case, the electrodes were mounted on a flat surface this configuration allows the measurement of the fluid level at the micron level without affecting the surface tension of the fluid.

**Figure 27:**
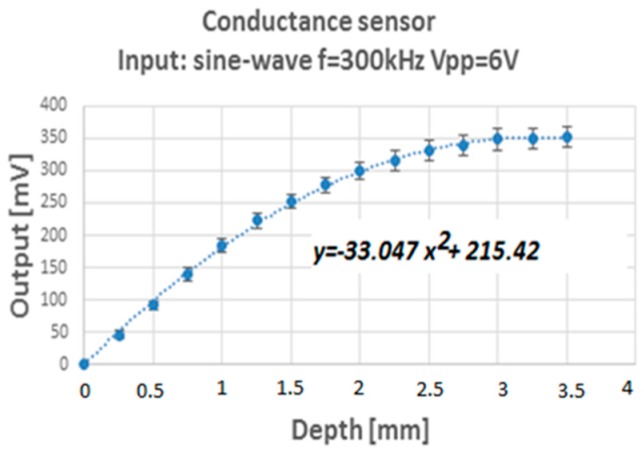
Values obtained with the calibration assembly.

Several measurements were performed varying the frequency and voltage of the excitation signal. The objective was to determine the values that provided the best voltage range of the output signal for the experimental water conditions (50 μS conductivity). The values obtained for the set of distance sweeps are shown in Figure 27.

Due to the operating characteristics of this type of sensors, both conductivity and fluid temperature are extremely important. However, according to literature, for a sufficiently narrow range of conductivities, the shape of the normalized voltage signal does not change appreciably.

**Figure 28:**
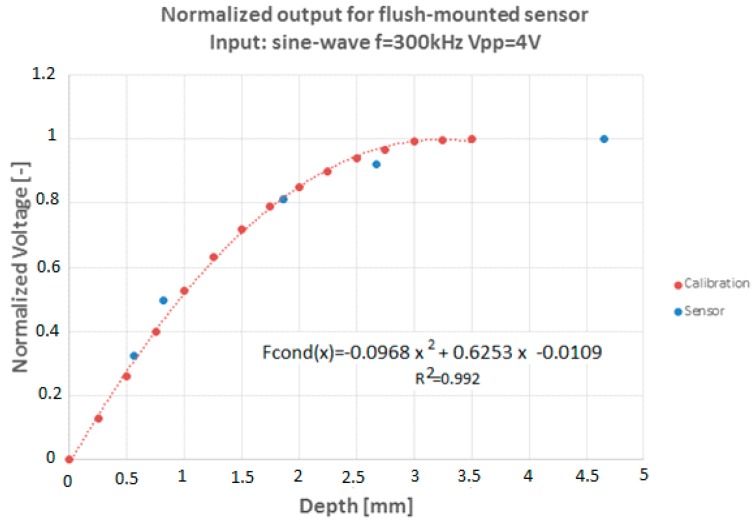
Normalized voltage values versus depth values.

Figure 28 displays the comparison between the calibration curve for flat geometry, i.e., the relation between signal values and the thickness values that were obtained with a flat plate, versus the calibration points obtained with the sensor mounted on a geometry with curvature as in the final assembly. The calibration curve with curvature was performed only for five points, performing the measurements with two concentric dielectric cylinders of previously known diameters; the radius of the inner cylinder made of a dielectric material was changed for the different calibration points. As can be seen both types of measurements give practically the same calibration curve.

Therefore, to obtain the liquid level it will be sufficient to use the normalized voltage scaled with $V_{liq}$ that is the voltage value corresponding to the saturation value of the sensor:

(40) $$Fcond\left(x\right)=\frac{V_{out}}{V_{liq}}$$

### 4.4. Preliminary Results

**Figure 29:**
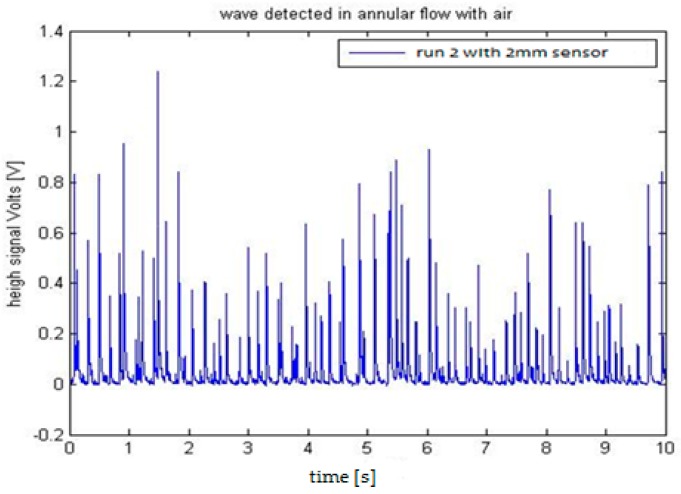
Signal obtained with 2 mm probe at VAFF facility at UPV.

**Figure 30:**
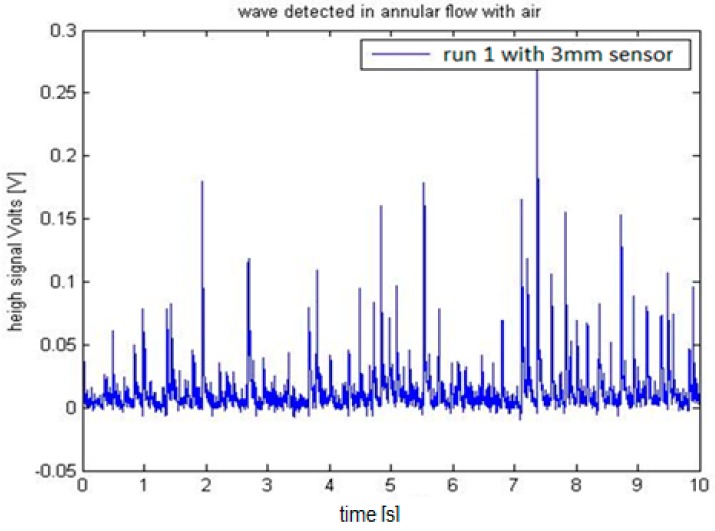
Signal obtained with the 3 mm probe at VAFF facility at UPV.

We installed the two sensors in the Vertical Annular Film Flow (VAFF) facility located in the Institute for Energy Engineering (IIE) of the Universitat Politècnica de València. The results obtained in the preliminary test were as expected, i.e., large amplitude waves called disturbance waves are observed followed by small waves known as ripples, and also when the sampling rate is increased to 100 kHz the disturbance waves had the typical shape predicted for film wave phenomena [35]. For the same annular flow conditions, in the sensor with 2 mm electrodes the acquired signal is greater than the signal recorded using the 3 mm sensor. One reason for this difference is that the surface for the 3 mm electrode is bigger than for the 2 mm one and the wave shape is averaged according to the electrode surface, producing signals of less amplitude. Another additional reason is that the distance s between the centers of the electrodes is bigger for the 3 mm sensor and the conductance diminish with this distance [32,33]. The signal to noise ratio was better with the 2 mm electrode than with the 3 mm electrode configuration. To accurately capture the shape of the wave, a sample rate of 100 kS/s was selected. The test conditions for the annular flow were a constant gas flowrate of 3900 L/min, a water flowrate of 4 L/min. The water temperature was 20 °C and the internal relative pressure of the pipe was 0.56 bars. Finally, we display in Figure 29 and Figure 30 the results obtained with both sensors.

The next step in the processing of the signal will be to filter the signal and to perform some signal analysis like Fast Fourier Transform, wavelets, etc., in order to better characterize all waves, both disturbances and ripples.

## 5. Discussion and Conclusions

Several conclusions are discussed; the first one is the quality of the sensors regarding corrosion, their manufacturing, and their overall performance. The second one is the validation of the conductivity probes and the methodology presented in this paper when we compare its results with the results of other authors. The third one is the validation performed comparing the results of the measurements obtained using the conductivity probes against the results obtained from other gauges. The main problem found during the manufacture of the multi-sensor conductivity probes was their fast degradation and corrosion of the conductivity probe tips and the isolation material that covers the needles. Therefore, we decided to investigate what was the best isolation material to cover the needles. This varnish should have a good dielectric behavior; also, an important property in the evaluation of this isolation material was that the selected material should be easy to deposit and form layers, which should be resistant to degradation by corrosion and hard enough to withstand the probe assembly process. We obtained the best results with urethane that showed a good resistance to corrosion and good dielectric material properties, and the layer by layer application was easy to perform. We selected for the sensor needles ones used in acupuncture, made of stainless steel and manufactured by Novasan (EnerQI CE0197) (Shangai, ,China). Then, we covered the non-sensing part with urethane as explained in Section 2.1, after that the coated needle remained four hours inside an oven at 60 °C. However, many trials with different materials and coatings were performed; Table 6 shows the performance of some of the varnished used to cover the needles as an example of the work performed to design each detail of the conductivity probe. One important question not discussed previously was the type of conductor cable used inside the sensors. We obtained the best results with a wire made of cupper coated with silver and with an isolating coating made of Kynar that is a fluoropolymer with good behavior against aging, corrosion, and with good mechanical resistance. The diameter selected was 0.25 mm, and is the one used actually. Another advantage is that permits to perform the small size welding required in the sensor.

The distance between the large tip and the short tips was chosen between 1.5 and 2 times the expected diameter of the bubbles, then for bubbly flow and the observed diameter of the bubbles, we used a distance of 2.2 mm. For the four-sensor conductivity probes as displayed in Figure 1a,b the shorter tips form an equilateral triangle of 0.45 mm each side, while the larger tip (red) is located at 0.4 mm from the nearest tip in the radial direction in one configuration and in the center of the equilateral triangle in the other one. In addition, we built conductivity probes with two tips. The characteristic of these conductivity probes with two sensors are displayed in Figure 2. Also in Table 1 the geometric characteristics of these conductivity probes are listed. However, we have not discussed the behavior of the probes F0X and F0A in previous sections. We have found, in the experiments performed using both types of sensors [10,11,12,13,20], that both types of sensors give similar information on the main parameters of the two-phase flow. However, the sensor F0A provides better information near the wall because the way in which the sensor tips are arranged allows locating the sensor F0A closer to the wall than the sensor F0X. In addition, the sensor F0X provides in general information of better quality than the sensor F0A when the sensor is used in regimes with relatively large bubbles, as could be the transition and slug flow regimes. This conclusion is a consequence of the analysis of the set of experimental data F, A, B and E performed at UPV.

An independent electric circuit fed each sensor tip and provided the current intensity and voltage needed by the conductivity probe, avoiding the electric coupling when using a parallel circuit for the tips. In addition, to have a good performance of the electric circuits was necessary to use highly stable and precise laboratory power-supply sources, by proceeding in this way the sensor signals were better conditioned and the data acquisition system received better quality signals. Figure 4 displays the prototype of the electric circuit used in the measurements at UPV and UJI.

To measure the two-phase flow characteristics, the conductivity probe was mounted inside the test channel with the needles parallel to the flow direction pointing downward. Then, the three sensor terminals SM, SC, and SL, for the two-sensor conductivity probe, were connected and the electric circuit was fed by the power supply with 5 Vdc, and a data acquisition frequency of 50 kHz. In order to positioning correctly the conductivity probe in the test section of the channel was necessary to design a device to bring the sensor into the test section avoiding fluid leakages and perturbing the two-phase flow as less as possible. Figure 5 displays this device denoted as “port”. In addition, we designed a device to move the sensor automatically to different radial positions, this device that contained a stepper electric motor controlled by computer is explained in Section 2.3.

The four and the two tip conductivity probes provided information about the time of residence of each phase in the sensors and about the time interval that needed each bubble to travel between the point where it touched the large tip and the point where it touched one of the shorter tips. To obtain this useful information from the signals was necessary to perform the signal processing that was executed in the following four steps: (i) signal conditioning, (ii) classification of the bubbles in groups, (iii) evaluation of the flow parameters, (iv) averaging and correction. All the procedures used for these steps are explained in Section 2.4, Section 2.7 and Section 2.8 and the methods of evaluation of the flow parameters and the averaging in Section 3. Finally, at Section 3.7 we display the experimental results of the F series and the boundary conditions for this series, discussing the results and if they are physically correct. Finally at Section 3.8 we explain the methods used to verify and validate that the method that we have used provide results that agree with the results obtained by other researchers when using the same boundary conditions [30,31]. Here a brief discussion is necessary. The comparison was performed with the data obtained by Hibiki et al. [30,31], using similar experimental boundary conditions than the ones used by this author. Although the results were very similar for the different physical magnitudes, as void fraction, Sauter mean diameter, interfacial area concentration and interfacial velocity as displayed in Figure 19 and Figure 20 they were not exactly the same because the water used had not the exactly the same properties of conductivity, PH, and dissolved salts. Otherwise, we used two types of four sensors conductivity probes in our measurements and Hibiki two-tip conductivity probes.

In addition to the previous cross-comparison to be completely sure that the results obtained with the sensors and the methodology used to obtain the physical magnitudes from the sensor signals was right, a set of crosschecking measures of the physical magnitudes of the two-phase flow was performed by different method as mentioned in Section 3.8. Here we discuss some of these crosschecking measurements. The void fraction at different heights was measured for the experimental conditions displayed in Table 4 and Table 5 using the sensor conductivity probe. In addition, the average void fraction was measured indirectly by means of pressure transducers, because the pressure at a given height of the channel depends on the average void fraction above the point where the pressure sensor is located and the pressure drop as we showed in Section 3.8. The correction due to the pressure drop at higher liquid superficial velocities and gas superficial velocities has two causes. First the increment in the liquid superficial velocity that increases the mass flow rate and therefore the pressure drop. Second the increments in the gas superficial velocity that increase the dynamic quality and therefore the two-phase multiplier increasing the pressure drop. At superficial velocities of the liquid phase of 0.5 m/s and 1 m/s, and low gas superficial velocities a good estimation of the void fraction can be obtained neglecting the pressure drop. However, for higher liquid and gas superficial velocities it is necessary to take into account the pressure drop to have good estimations of the void fraction. In Figure 21a–d we display the results obtained for the void fraction using both conductivity probes and manometers at liquid superficial velocities of $j_{f}$ = 0.5, 1, 2 and 3 m/s. We observed that most of the data are within the ±10% band or close to this band. Only for the cases F0X4 and F0A4 with the highest liquid superficial velocities, it is observed some dispersion, but even in these cases, the results are good.

Figure 22 illustrates the cross check performed comparing the volumetric gas flow rate injected in the water column and measured with an air flow meter with the volumetric gas flow rate measured using the multi-sensor conductivity probe. Most of the results are within the error band ±10%. Therefore, we can say that the sensor and the methodology explained in this paper to obtain the two-phase flow parameters give in general good results.

Also in Section 4, we showed the main characteristics of the sensors designed to study the interfacial waves in annular flow. Two different calibration methods have been used for the conductance probes, both methods agree, and two different types of conductance sensors have been designed.

## Figures and Tables

**Table 1:** Main geometric characteristic of the two-sensor conductivity probes prototypes.

Characteristics	Values [mm] Prot-1	Values [mm] Prot-2
Dp	3	3
P	250	240
Dc	2.5	1.2
Lc	22	60
S	1	0.5
L	3	20
X	1.5	2.2

**Table 2:** Measurements of the electric resistance of the fluid medium performed at UPV [20].

Variable	Water Value	Units	Variable	Air Value	Units
*V*	5.07	Vdc	V	5.07	Vdc
$R_{V}$	50.5	kΩ	Rv	50.5	kΩ
*R*	99.1	kΩ	R	99.1	kΩ
$R_{m}$	55.45	kΩ	Rm	28.75	MΩ
*I*	22.5	μA	I	0.16	μA

**Table 3:** Comparison between the superficial velocities obtained by LDA and the electromagnetic flow-meter.

Void Fraction	$Q_{l}/A$	$Q_{l,emf}/A$	Difference%
5%	0.498	0.513	−2.946
10%	0.482	0.516	−6.677
15%	0.481	0.519	−7.337
20%	0.483	0.521	−7.141
23%	0.487	0.523	−6.941
25%	0.535	0.525	2.023

**Table 4:** Experimental flow conditions for the experiments performed with sensor F0X.

*j_f_* = 0.51 m/s			*j_f_* = 1.023 m/s			*j_f_* = 2.036 m/s			*j_f_* = 3.086 m/s		
	*j_g_* [m/s]	<α>_z/d=sup_ [−]		*j_g_* [m/s]	<α>_z/d=sup_ [−]		*j_g_* [m/s]	<α>_z/d=sup_ [−]		*j_g_* [m/s]	<α>_z/d=sup_ [−]
**F0X1G00**	0	0	**F0X2G00**	0	0	**F0X3G00**	0	0	**F0X4G00**	0	0
**F0X1G01**	0.035	5.56	**F0X2G01**	0.058	5.44	**F0X3G01**	0.097	5.20	**F0X4G01**	0.166	4.54
**F0X1G02**	0.077	10.18	**F0X2G02**	0.142	10.92	**F0X3G02**	0.233	11.46	**F0X4G02**	0.389	7.48
**F0X1G03**	0.125	15.34	**F0X2G03**	0.235	15.27	**F0X3G03**	0.47	15.23	**F0X4G03**	0.662	12.16
**F0X1G04**	0.176	20.32	**F0X2G04**	0.396	19.90	**F0X3G04**	0.72	24.08	**F0X4G04**	1.023	15.60
**F0X1G05**	0.257	22.22	**F0X2G05**	0.67	26.10	**F0X3G05**	1.181	28.14	**F0X4G05**	1.695	19.60
**F0X1G06**	0.338	24.73									

**Table 5:** Experimental flow conditions for the experiments performed with sensor F0A.

*j_f_* = 0.506 m/s			*j_f_* = 1.027 m/s			*j_f_* = 2.026 m/s			*j_f_* = 3.033 m/s		
	*j_g_* [m/s]	<α>_z/d=sup_ [−]		*j_g_* [m/s]	<α>_z/d=sup_ [−]		*j_g_* [m/s]	<α>_z/d=sup_ [−]		*j_g_* [m/s]	<α>_z/d=sup_ [−]
**F0A1G00**	0	0	**F0A2G00**	0	0	**F0A3G00**	0	0	**F0A4G00**	0	0
**F0A1G01**	0.035	5.42	**F0A2G01**	0.059	5.36	**F0A3G01**	0.098	4.18	**F0A4G01**	0.166	3.30
**F0A1G02**	0.077	10.42	**F0A2G02**	0.141	10.6	**F0A3G02**	0.228	10.13	**F0A4G02**	0.396	7.43
**F0A1G03**	0.125	15.75	**F0A2G03**	0.235	15.5	**F0A3G03**	0.474	15.73	**F0A4G03**	0.668	11.6
**F0A1G04**	0.174	20.25	**F0A2G04**	0.361	20.58	**F0A3G04**	0.727	23.34	**F0A4G04**	1.037	16.5
**F0A1G05**	0.256	21.8	**F0A2G05**	0.676	26.98	**F0A3G05**	1.202	27.98	**F0A4G05**	1.738	19.95
**F0A1G06**	0.406	28.04									

**Table 6:** Results of the proof performed by Mendez at UPV with different varnish of the sensor needles.

Coating Type	Layer Deposition Performance	Humidity Resistance	Dielectric Behaviour
Royalac 128	Good	Medium	Good
Plastik 70	Bad	Good	Good
Urethane	Good	Good	Good
Polyurethane RS	Medium	Medium	Medium
Aropol	Bad	Good	Good
Varnish RS	Good	Bad	Medium
